# Identifying risk clusters for African swine fever in Korea by developing statistical models

**DOI:** 10.3389/fvets.2024.1416862

**Published:** 2024-07-24

**Authors:** Kyeong Tae Ko, Janghun Oh, Changdae Son, Yongin Choi, Hyojung Lee

**Affiliations:** ^1^Department of Statistics, Kyungpook National University, Daegu, Republic of Korea; ^2^Busan Center for Medical Mathematics, National Institute for Mathematical Sciences, Daejeon, Republic of Korea

**Keywords:** African swine fever, spatial dynamics, temporal heterogeneity, generalized linear model, statistical modeling, risk clusters

## Abstract

**Introduction:**

African swine fever (ASF) is a disease with a high mortality rate and high transmissibility. Identifying high-risk clusters and understanding the transmission characteristics of ASF in advance are essential for preventing its spread in a short period of time. This study investigated the spatial and temporal heterogeneity of ASF in the Republic of Korea by analyzing surveillance data on wild boar carcasses.

**Methods:**

We observed a distinct annual propagation pattern, with the occurrence of ASF-infected carcasses trending southward over time. We developed a rank-based statistical model to evaluate risk by estimating the average weekly number of carcasses per district over time, allowing us to analyze and identify risk clusters of ASF. We conducted an analysis to identify risk clusters for two distinct periods, Late 2022 and Early 2023, utilizing data from ASF-infected carcasses. To address the underestimation of risk and observation error due to incomplete surveillance data, we estimated the number of ASF-infected individuals and accounted for observation error via different surveillance intensities.

**Results:**

As a result, in Late 2022, the risk clusters identified by observed and estimated number of ASF-infected carcasses were almost identical, particularly in the northwestern Gyeongbuk region, north Chungbuk region, and southwestern Gangwon region. In Early 2023, we observed a similar pattern with numerous risk clusters identified in the same regions as in Late 2022.

**Discussion:**

This approach enhances our understanding of ASF spatial dynamics. Additionally, it contributes to the epidemiology and study of animal infectious diseases by highlighting areas requiring urgent and focused intervention. By providing crucial data for the targeted allocation of resources for disease management and preventive measures, our findings lay vital groundwork for improving ASF management strategies, ultimately aiding in the containment and control of this devastating disease.

## 1 Introduction

African swine fever (ASF) is a severe viral infection that causes hemorrhagic fever in pigs, often leading to a high fatality rate of approximately 100% (1). According to the World Organization for Animal Health (WOAH) (2), ASF is a priority disease due to its significant health and economic repercussions for swine producers and government disease control agencies. ASF infection is caused by the African swine fever virus (ASFV), which belongs to the genus *Asfivirus*, and can be transmitted through both direct and indirect pathways. Direct transmission occurs through contact with the live bodies or carcasses of infected pigs, while indirect transmission happens via contact with contaminated objects, such as feed, water, and needles (3, 4). Some studies have utilized Geographic Information Systems (GIS) and remote sensing technologies to analyze disease spread in wildlife based on environmental factors and spatial data, confirming their potential role in monitoring and management (5–8). These studies have indicated that ASF spread can vary according to spatial characteristics.

The ASF outbreak was first documented in Kenya in 1921 (9) and became endemic in some regions of Africa. Subsequently, it spread to Europe and South America, where it was mostly eradicated. However, in 2007, the virus was introduced to Europe through Georgia (10), leading to widespread transmission. In Asia, the first case was reported in China in August 2018, followed by occurrences in other Asian countries (11–14). ASFV is typically classified based on pathogenicity into high, moderate, and low virulence. Highly virulent strains cause death within approximately 8 days, moderately virulent strains within about 20 days, and low virulence strains result in subclinical or chronic disease (4, 15). Chronically infected individuals play a crucial role in the long-term persistence of the virus, making early eradication difficult (16).

In the Republic of Korea, the first confirmed case was identified in September 2019 at a pig farm in Paju, Gyeonggi Province, and it has since continued to spread, primarily in the Gyeongbuk region (17, 18). Between October 9, 2019, and May 20, 2024, approximately 3,555 cases have been reported, involving 40 domestic pig farms and 3,515 wild boars (19, 20). Low virulence strains of ASFV have been predominantly identified in endemic regions such as Northern Europe and China. In China, the emergence of chronically infected individuals has been attributed to the production and use of illegal vaccines (21). Conversely, ASF outbreaks in the Republic of Korea have been confirmed to be caused by highly virulent strains, leading to death within 8–10 days post-infection (22, 23). Based on this information, we consider that ASF-infected carcasses reveal the overall spread patterns of ASF infection in the Republic of Korea, though they may not perfectly reflect real-time infection trends. This assumption is supported by previous studies, which also utilized carcass data to identify risk clusters for ASF outbreaks in the Republic of Korea (24).

Another characteristic of ASF spread in the Republic of Korea is that direct transmission of ASF between wild boars and domestic pigs is relatively unlikely because domestic pigs are confined in enclosed pigsties within fenced buildings, such as intensive indoor housing (25). However, ASF transmission to domestic pigs is possible via objects associated with ASF-infected wild boar or human interactions (3, 26). Accordingly, the Korean government implemented systematic and comprehensive interventions to prevent the spread of ASF, including reducing the density of wild boars, promptly disposing of carcasses, and installing fences around ASF-infection areas (27–29). To further preemptively block the spread of ASF between regions, extensive fencing was installed in six phases (stage 1, stage 2, …, stage 5–1, stage 5–2) from November 2019 to May 2022, spanning a total of 1,831 kilometers across 34 districts (3, 17, 30, 31). Several studies have confirmed the importance of active preventive measures, including the installation of fences (29, 32, 33). Notably, a study by Lim et al. (32) showed that the third phase of national fencing decreased the infection pressure on individuals in neighboring habitats by 47% compared to the same geographical habitat. This finding supports the effectiveness of fencing in limiting wild boar movement and reducing ASF transmission. However, despite these interventions and achievements, outbreaks in pig farms and wild boar populations have not been completely controlled. The continued occurrence of ASF highlights the need for a deeper understanding of the mechanisms of disease spread, emphasizing the necessity of predicting and analyzing risk clusters to improve ASF control strategies.

To understand the mechanisms of ASF spread, many studies have been conducted on factors influencing disease transmission, including the environmental and geographical factors. An analysis of the impact of environmental factors on ASF outbreaks and control revealed that the presence of roads and rivers effectively reduces the transmission rate by approximately 37% on average (34). Additionally, wild boars living at altitude above 1,000 meters are difficult to control through hunting, and the probability of transmission is higher in certain forest areas, such as the Taebaek Mountains across the Republic of Korea and the Democratic People’s Republic of Korea (32). In the present study, we aimed to analyze the patterns of ASF outbreaks using the geographical coordinates (longitude and latitude) of ASF-infected carcasses, along with spatial information such as forest area, slope, and altitude. By estimating the risk clusters for emerging ASF outbreaks, this study could provide alternative approaches for developing surveillance systems.

## 2 Materials and methods

**Figure 1:**
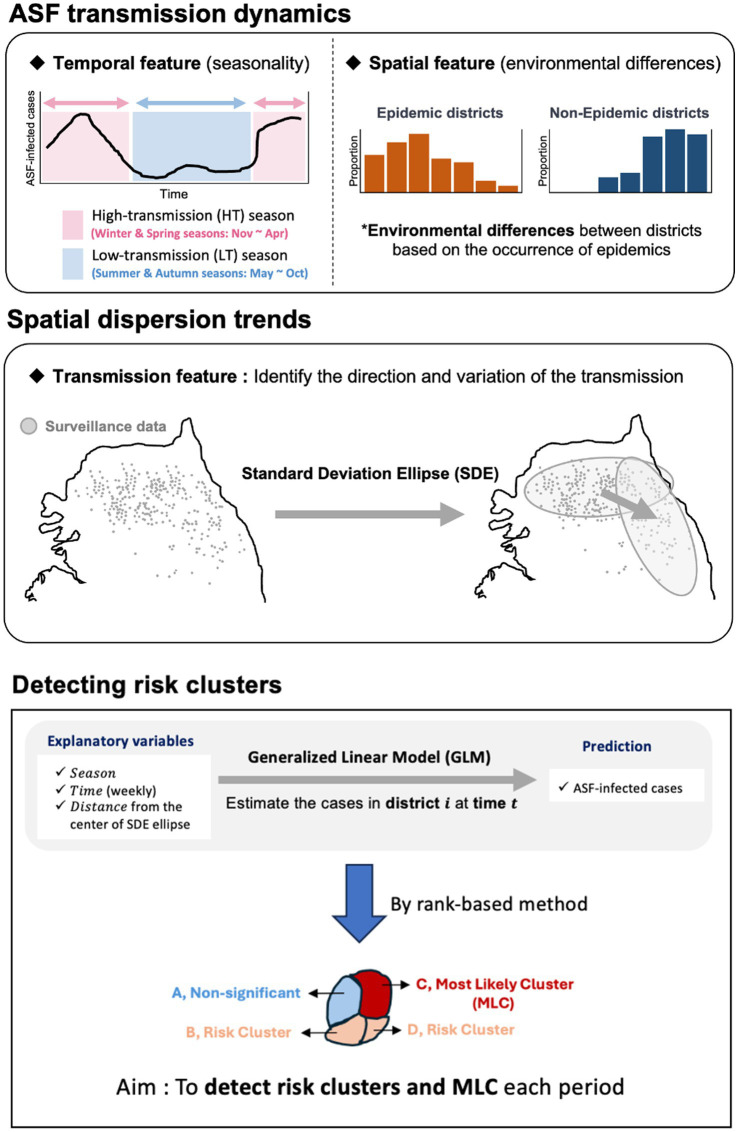
An outline for identifying risk clusters and most likely clusters (MLC) using rank-based score.

We conducted a statistical analysis to identify risk clusters under surveillance for ASF outbreaks by investigating the continually evolving spreading patterns of ASF based on information on ASF-infected carcasses reported in the Republic of Korea from October 2019 to April 2023. Using Standard Deviation Ellipse (SDE) analysis and rank-based method, we aimed to identify regions at risk of ASF outbreaks. Figure 1 illustrates the statistical analysis approach used to identify risk clusters. We assessed the direction and variance of the spread using the SDE to comprehend the spatial transmission patterns at each time point. Furthermore, we constructed a generalized linear model (GLM) to compute the number of wild boar carcasses in the region for each period using the designated probability distribution. We developed a statistical model using a rank-based method to evaluate ASF risk. This model estimates the average weekly number of carcasses by district over time, allowing us to analyze and identify risk clusters. To mitigate the underestimation of risk and account for observation errors caused by undetected ASF-infected individuals, we estimated the number of ASF-infected animals and incorporated observation error by considering different surveillance intensities.

### 2.1 Data analysis

#### 2.1.1 Data description

**Figure 2:**
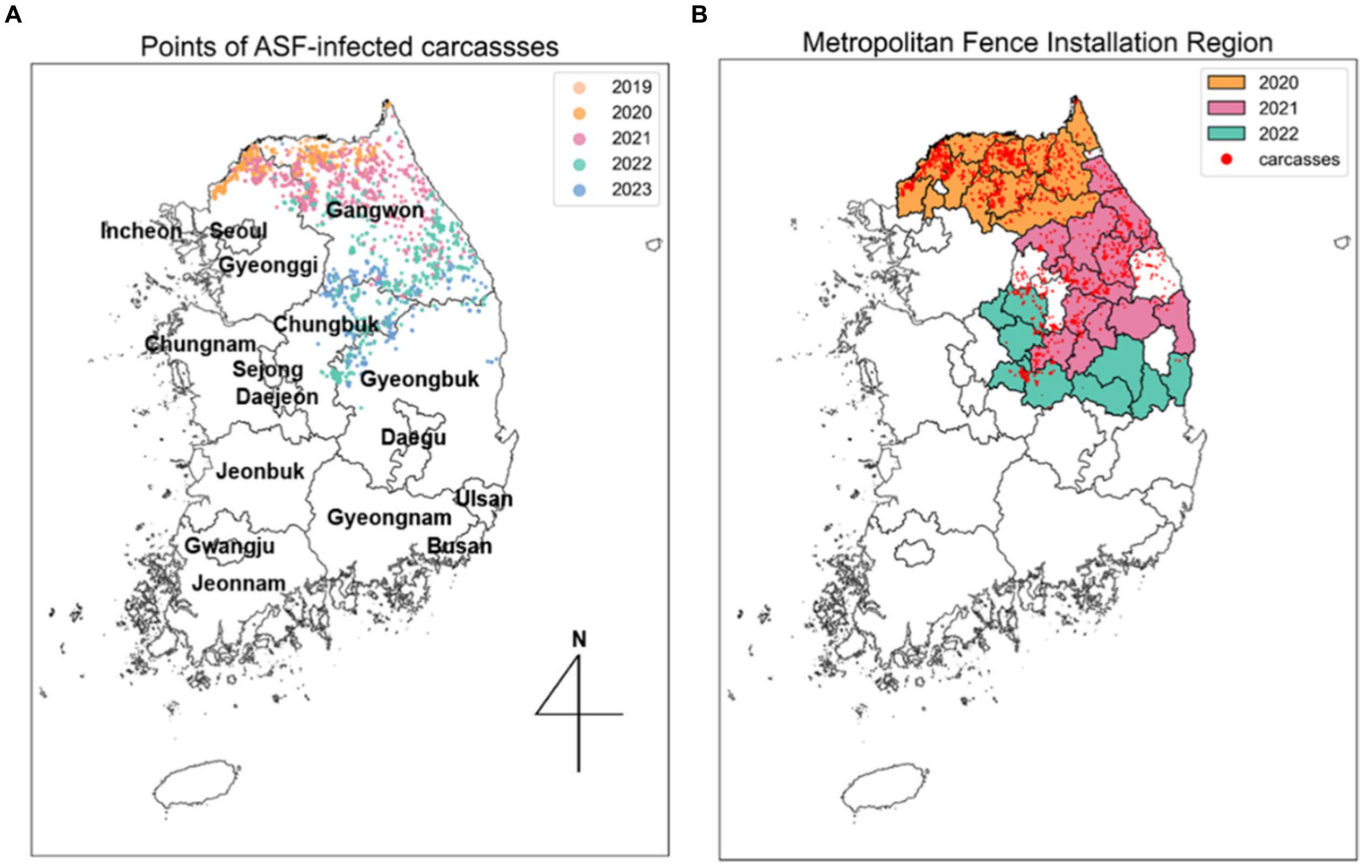
Spread trend of ASF outbreaks from SDE. **(A)** Five different colors indicate the ellipses obtained from SDE by year. Points, representing the observed locations of carcasses for each year, are colored to match the corresponding year’s ellipse. **(B)** Each colored area outlines the regions where metropolitan fences were installed each year, corresponding to the same color scheme used for the ellipses.

The spatial distribution of this surveillance data is illustrated in Figure 2A. The surveillance data on wild boar carcasses infected with ASFV from October 2019 to April 2023 in the Republic of Korea was analyzed. This dataset, compiled from data provided by the Ministry of Agriculture, Food, and Rural Affairs (MAFRA) (20) and supplemented with information from the ASF real-time status board derived from the *Google Map* service (35), includes diagnosis dates, observation dates, observation methods (including carcass removal and hunting), geographical coordinates (longitude and latitude), and the locations of the collected samples. Observation dates denote when carcasses were discovered, while diagnosis dates indicate the confirmation of an ASFV-positive diagnosis.

The administrative boundary data in the Republic of Korea comprises approximately 250 districts and 17 regions. In this study, a “district” is defined as a city, county, and borough, while “area” refers to several cities. To analyze the trends in the spread of ASF, we collected information on the installation of extensive fencing and environmental data, including forest area ( $m^{2}$ ), elevation (altitude), and slope data by district, all classified by administrative boundaries. Extensive fencing data, sourced from the Ministry of Environment (17), indicates that 1,831 kilometers of fencing were installed across 34 districts. To interpret the effectiveness of these fencing control measures, we categorized the areas with additional fencing installed annually from 2020 to 2022 and organized this along with the ASF-infected carcass data, as shown in Figure 2B. The ASF-infected carcasses data and environmental data for Gyeonggi and Gangwon regions are summarized in Supplementary Table S1.

#### 2.1.2 Estimation of ASF-infected counts from ASF-infected carcass data

ASF-infected carcass information serves as useful indicators of infection spread and is therefore used as primary data. Many previous studies have utilized ASF-infected carcass data to understand the dynamics of ASF (18, 36, 37). However, due to potential underestimation of the outbreak scale caused by delays in carcass detection and the period between becoming infectious and death, ASF-infected carcass data may be insufficient to accurately reflect the actual spread of infection. Thus, we estimated the number of infected individuals (estI) over time and used this estimation as comparative data to the observed number of ASF-infected carcasses (obsC) to more accurately represent true infections that could impact actual transmission.

ASFV found in the Republic of Korea from 2019 to 2023 is typically highly pathogenic, causing death in infected pigs within a week (18, 23). Infection experiments conducted in the Republic of Korea revealed that the estimated time to virus detection was 3.7–4.8 days, the incubation period was between 3.4 and 5.2 days, and the time to death was 8.9 to 9.1 days (38). Based on these findings, we assume that the time from infection to carcass discovery follows a uniform distribution with a mean of 9 days and a range between 0 and 18 days. Throughout the entire time, we estimated estI by back-calculating from the obsC. This process involved estimating the time interval between the observation dates of obsC and the estimated infection dates, which were randomly generated from a Uniform (0, 18) distribution, allowing us to estimate estI over time. We compared the risk clusters identified using obsC and estI to verify differences in risk assessment. This comparative approach is expected to preemptively identify risk clusters and enable more proactive predictions compared to relying solely on carcass-based observations.

### 2.2 Spatial and temporal dynamics of ASF transmission

Observational data have revealed that ASFV infections are more prevalent during the winter and spring seasons, which coincide with the wild boar breeding season (39), contrasting with lower frequencies of infections observed in summer and autumn (32). Accordingly, we have stratified the year into two distinct seasons for analysis: the “high transmission season (HT season),” spanning from November to April, and the “low transmission season (LT season).” This division allows for a systematic investigation of the temporal heterogeneity and transmission dynamics of ASF. The distinction is supported by the observation that about 80% of ASF cases in the Republic of Korea occurred during the HT season from 2020 to 2022. Additionally, we categorized the 250 districts into two groups: epidemic districts, where ASF cases have been reported, and non-epidemic districts, where no cases have been reported. This analysis particularly focused on the Gyeonggi and Gangwon regions, which account for over 80% of the carcass count.

We conducted statistical hypothesis tests to analyze temporal variations in carcass counts between HT and LT seasons and distributional differences of environmental factors such as forest area, elevation, slope between epidemic and non-epidemic districts.

First, we employed the augmented Dickey–Fuller (ADF) test to examine the stationarity of cumulative ASF-infected carcasses during the HT and LT seasons. The ADF test serves as a statistical tool to determine the stationarity of time-series data based on the null hypothesis that stationary data do not maintain constant statistical properties over time (40). This approach is particularly relevant for identifying temporal variations in ASF transmission rates, offering a statistical basis to assess the effects of seasonality on the spread of ASF.

Second, we applied non-parametric tests including the Ansari–Bradley and Mann–Whitney U tests to investigate distributional differences in forest area, slope, and elevation (altitude) between the epidemic and non-epidemic districts. Higher *p*-values from the Ansari–Bradley test indicate variability in dispersion patterns, while lower *p*-values from the Mann–Whitney U test highlight significant differences in central tendencies. These tests were selected because of their efficacy in managing the nonnormal distribution of data, thereby enabling a robust comparison of the variances and median values between the two district groups (41, 42).

Through this methodological approach, we aimed to enrich the comprehension of the spatial and temporal analysis conducted in our study, subsequently providing insights into the unique transmission characteristics of ASF in the Republic of Korea, described within specific seasonal and geographical contexts.

### 2.3 Statistical modeling for estimating the number of carcasses

#### 2.3.1 Standard deviation ellipse

The SDE is a spatial statistical tool widely used to describe the directional trend and dispersion of geographical features in spatial distribution, utilizing the longitudinal and latitudinal locations of ASF-infected carcasses (43, 44). Historically, SDE has been used to analyze spatial dispersion and directional bias in Poland (45) and to investigate the directional trend and spread of Foot and Mouth Disease (FMD) in China (46). Building on these applications, we employed the SDE method using observed carcass data to identify annual and monthly changes in occurrence regions of ASF in the Republic of Korea. This method facilitates the creation of ellipses that capture spatial characteristics such as orientation, spatial dispersion, and directional trends of ASF outbreaks.

The outcomes of the SDE method include the lengths of longitudinal and latitudinal axes, their ratio, angle, and the center point. The lengths of the longitudinal and latitudinal axes quantify the dispersion in the east–west (horizontal) and north–south (vertical) directions, respectively, based on the variance of the obsC data used for measurement. Moreover, the angle of the ellipse, determined by the longer axis, indicates the principal direction of data spread, starting from north and moving clockwise. This reveals the direction in which the spatial dispersion pattern of the data tends. The SDE ratio, a longitude-to-latitude measure, indicates the ellipse’s deviation from a circular shape based on the lengths of the longitudinal and latitudinal axes (44). A ratio between zero and one indicates a vertical dispersion tendency, whereas a ratio greater than 1 implies a horizontal dispersion tendency. As the axis lengths approach equality, nearing a ratio of one, the ellipse tends to resemble a circle, suggesting limited propagation in a specific direction.

The dimensions of these ellipses, represented by their long and short axes, are defined by the variances in longitude and latitude of the observed carcasses. The size of the ellipse, adjustable based on the carcass count, is governed by the variance in both dimensions (47, 48), and the angle of the ellipse is derived from the covariance between longitude and latitude. Using sigma ( σ ) to denote the standard deviation for both longitude and latitude, a single sigma ( 1σ ) along each axis typically encompasses approximately 66.7% of all carcasses within the ellipse. Two sigma ( 2σ ) captures about 95.5%, and three sigma (3 σ ) includes approximately 99.7% of the total carcasses (48, 49).

#### 2.3.2 Effect of the surveillance intensity on ASF-infected carcasses

Surveillance data on ASF-infected carcasses is collected through a government-implemented carcass collection policy, and this data is crucial for analyzing the response to the ASF outbreaks. Typically, surveillance and control policies, such as installing fences to prevent spread, are initiated in districts where ASF-infected carcasses are found (17). Considering the limited resources available for response policies, the intensity of response may vary according to the outbreak severity in different areas. It is expected that surveillance will be intensified in districts with a higher number of ASF-infected carcasses discovered. Conversely, districts with lower surveillance intensity are likely to have a higher number of undiscovered infected carcasses. This difference between the actual and reported number of infected individuals, defined as the observation error, can vary with the surveillance intensity.

To understand the impact of surveillance intensity, we conducted a scenario analysis focused on observation errors. We employed a spatial dispersion analysis of ASF spread using SDE analysis of ASF-infected carcass data collected during the observation period to delineate surveillance areas. Surveillance intensities were adjusted across different zones of the ellipse based on the standard deviation (sigma) setting. These zones included 66.7% of the data within the 1-sigma ellipse, 95.5% within the 2-sigma ellipse, and 99.7% within the 3-sigma ellipse, with surveillance intensity decreasing progressively from the 1-sigma to the 3-sigma ellipse.

We calculated the adjusted number of ASF-infected carcasses ( $adjC_{i,t}$ ) in district *i* at time *t* from the $obsC_{i,t}$ through different observation error rates ( ε% ), determined by the intensities of surveillance, as follows:


$$adjC_{i,t}=obsC_{i,t}\left(1+\varepsilon /100\right)\text{.}$$

The $obsC_{i,t}$ are set according to designated observation error rates at each stage of surveillance intensity as follows:

- **(Strong intensity)** In 1-sigma districts, the surveillance is conducted at maximum intensity with an observation error of 0%, implying that $adjC_{i,t}$ equals the observed number:

$$adjC_{i,t}=obsC_{i,t}$$

- **(Intermediate intensity)** In 2-sigma districts, the observation error is defined by a specific factor, adjusting the observed number to estimate the total number as:

$$adjC_{i,t}=obsC_{i,t}\left(1+\varepsilon /100\right)$$

- **(Low intensity)** In 3-sigma districts, the observation error is twice that of 2-sigma districts:

$$adjC_{i,t}=obsC_{i,t}\left(1+2\;\varepsilon /100\right)$$

- **(Not implemented)** Surveillance is not implemented outside the 3-sigma ellipse, where ASF occurrences are negligible, covering only 0.3% of the observation data.

### 2.4 Analysis of the generalized linear model

We employed a GLM to estimate the weekly number of ASF-infected carcasses across 250 districts in the Republic of Korea over time. The GLM extends the linear regression model to accommodate response variables following various probability distributions, such as Poisson, Negative binomial, and Zero-inflated distributions, by connecting them with the response variable through a link function, $f\left(\cdot \right)$ . In our model, we assumed the number of carcasses, $Y_{\mathrm{it}}$ in district *i* at time *t*, adheres to these designated distributions. The GLM to estimate the number of ASF-infected carcasses ( $estC_{i,t}$ ) in district i at time t , is formulated as:


$$\begin{array}{l} f\left(Y_{\mathrm{it}}\right)=\alpha +\beta_{1}\;\mathrm{Distanc}e_{i,t}+\beta_{2}\mathrm{Distanc}{e_{i,t}}^{2}+\\ \beta_{3}\;\mathrm{Seaso}n_{t}+\beta_{4}\;Time_{t} \end{array}$$

Where $\beta_{1},\beta_{2},\beta_{3}\;\mathrm{and}\;\beta_{4}$ represent the coefficients for each variable, and α is the intercept. The outcomes of the SDE analysis revealed spatial features that were previously unconsidered in the regression equation. The Distance variable represents an exponential decay of the Euclidean distance, thereby assigning higher transmission risk to closer districts. By incorporating these insights, we defined the *Distance* variable for district i at time t as $\mathrm{Distanc}e_{i,t}=\exp\left(-d_{ij}\left(t\right)\right)$ . Here, $d_{ij}\left(t\right)$ represents the Euclidean distance between district i and the center point j of the SDE ellipse at time t . j is defined as the center point of the SDE ellipse using carcasses data from 2022 if t∈2022 or using carcasses data from 2023 if t∈2023. To capture any non-linear relationships between geographical distance and obsC, we also introduced the $\mathrm{Distanc}e^{2}$ variable.

The *Season* variable serves as an indicator variable to distinguish between HT and LT seasons, reflecting seasonal variations in ASF occurrence. Additionally, the *Time* variable is introduced to track the progression of weeks during the study period, starting from one and sequentially increasing, which helps incorporate temporal dynamics into our analysis (i.e., *Time* = 1, …, 16 for Late 2022 and *Time* = 1, …, 17 for Early 2023). By integrating these variables, our model captures the heterogeneity of ASF dynamics across different geographical regions and over time. This comprehensive approach enhances our ability to predict ASF spread more accurately in the Republic of Korea.

This approach, supported by previous research (50), assumes that carcass counts follow designated distributions, with Poisson regression widely used in epidemiology for analyzing count data (51, 52). However, the Poisson distribution’s assumption of equal mean and variance may not always align with real-world data, prompting us to include models based on Negative Binomial (NB) and Zero-Inflated Poisson (ZIP) distributions. This expansion, inspired by prior research including studies on pig infection counts in Sardinia (51), aims to overcome the limitations of the Poisson model. Our analysis suggests that outcomes can vary depending on the model employed to estimate the ASF-infected carcass counts. This variability allows for a range of estimation outcomes influenced by the differential impact of diverse distributions on the coefficients of the independent variables. To facilitate this analysis, we generated the estimates from each distribution according to the estimated coefficients, thereby enabling a comparison. This approach offers a comprehensive analysis of ASF outbreak data using various distributions and provides a deeper understanding of the dynamics of ASF outbreaks.

This analysis was implemented using the R programming language. To fit the GLM models, we used the “glm” function from the “stats” package for Poisson distributions, the “glm.nb” function from the “MASS” package for NB distributions, and the “hurdle” function from the “pscl” package for a ZIP distributions.

### 2.5 Identifying risk clusters of ASF outbreaks

**Figure 3:**
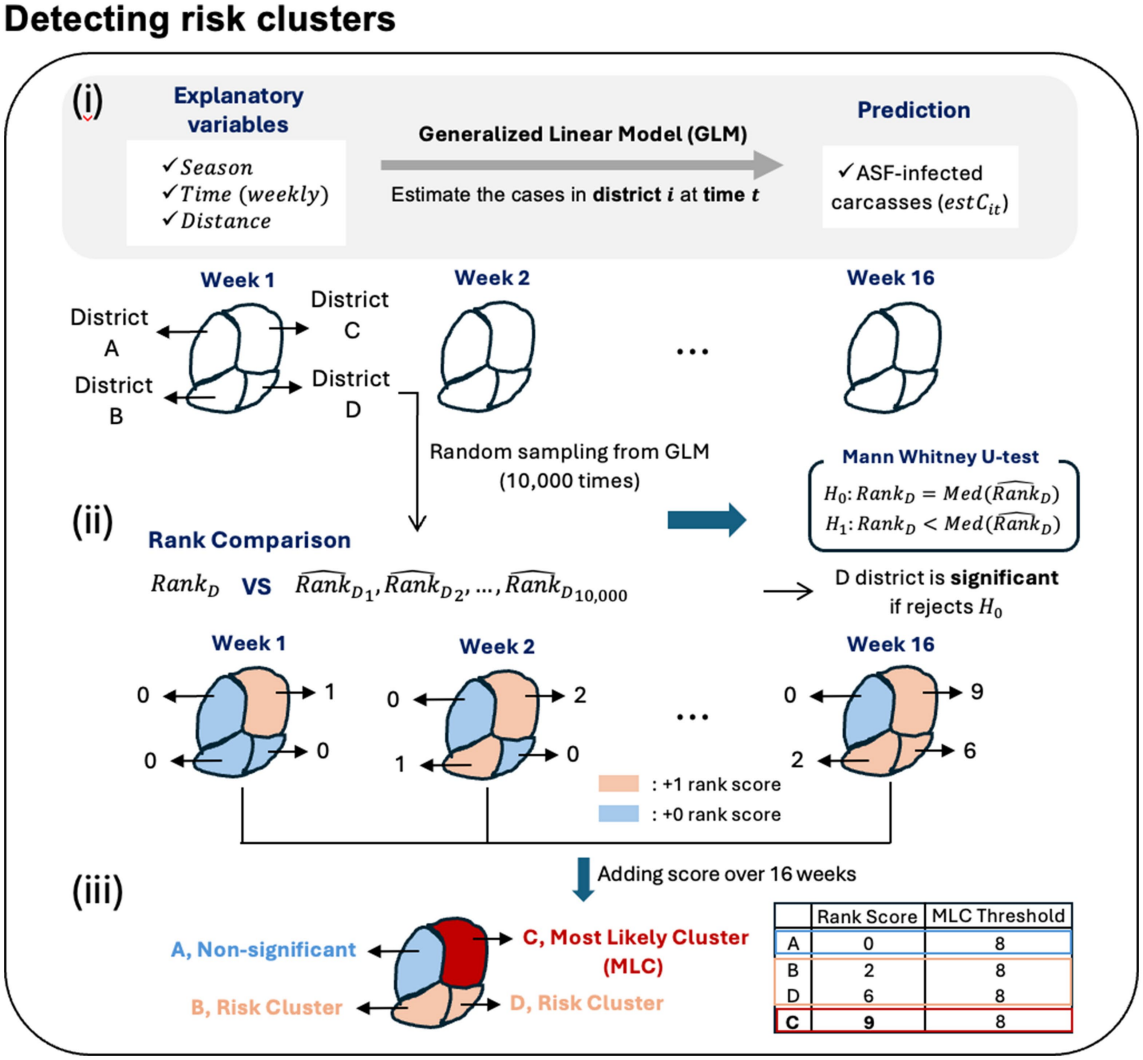
Outline for detecting the risk clusters of ASF outbreaks.

We conducted our analysis during the HT period to identify risk clusters for two periods: September to December 2022 (Late 2022) and January to April 2023 (Early 2023). When ASF occurrence in a specific area surpasses the GLM-based estimate, it suggests either randomness or a higher than expected level of occurrence, signaling the need for heightened attention to that area. However, since surveillance data is collected based on the discovery of carcasses of infected individuals rather than the real-time number of infected cases, identifying risk clusters by simply comparing the surveillance data with the estimated counts is less effective. Instead of focusing solely on carcass counts, we compared the rankings of areas with a high risk of ASF occurrence between observed data and estimates from GLM. Specifically, we ranked areas based on the obsC over a week to identify regions with high outbreak risk, while simultaneously ranking areas based on predicted carcass counts to determine expected risk districts. Furthermore, we used the Mann–Whitney U test, also known as the Wilcoxon rank-sum test, which is a non-parametric method for comparing the medians between two groups, to evaluate whether the actual rank is significantly higher than the estimates. Subsequently, we defined a risk cluster as one where the rank of the actual risk cluster is significantly higher than the rank of the expected risk cluster. We elucidated the following procedures to identify risk clusters for ASF outbreaks, described in Figure 3:

- Estimation of the ASF case counts using GLM: Given the observed number of ASF-infected carcasses ( $obsC_{i,t}$ ), we estimated the number of ASF-infected carcasses ( $estC_{i,t}$ ) in district i and week t , where i = 1, …, 250 and t = 1, …, 16 for Late 2022 and t = 1, …, 17 for Early 2023. Here, we assumed that ASF-infected carcass counts follow a designated distribution. To ensure the robustness of our estimates, we generated 10,000 random samples ${s_{i,t}}^{\left(1\right)},\cdots ,{s_{i,t}}^{\left(10000\right)}$ from the designated distribution with parameters $\lambda_{i,t}$ for each district *i* and time *t*.
- Rank score by Rank-based method and Mann Whitney U-test: We assigned ranks to each of the 250 districts based on the $obsC_{i,t}$ for each week t , denoted by $R_{1,t},\cdots ,R_{250,t}$ . Similarly, for all samples, we assigned ranks across the 250 districts through comparison. For example, in the case of the n th sample across the 250 districts, ${s_{1,t}}^{\left(n\right)},\cdots ,{s_{250,t}}^{\left(n\right)}$ , assigned ranks included ${r_{1,t}}^{\left(n\right)}$ , …, ${r_{250,t}}^{\left(n\right)}$ . Subsequently, we calculated the median rank for each district based on the ranks assigned to all samples, with the median rank for district i and week t denoted as $\hat{R_{i,t}}$ . In other words, the ranks generated for each sample in district comparison for district i and week t , resulting in a total of 10,000 ranks ${r_{i,t}}^{\left(1\right)}$ , …, ${r_{i,t}}^{\left(10000\right)}$ , are used to find the median value as $\hat{R_{i,t}}$ . Based on the measured ranks for a specific district *i* and time *t*, $R_{i,t}$ and $\hat{R_{i,t}}$ , we constructed the null hypothesis as $H_{0}:R_{i,t}=\hat{R_{i,t}}$ and the alternative hypothesis as $H_{1}:R_{i,t}<\hat{R_{i,t}}$ . Then, we could determine the significance by examining the *p*-values. If the *p*-value is less than 0.05, it is concluded that the actual carcass rank is significantly higher than the estimated carcass rank.
- Risk clusters and the most likely clusters: The rank-based score (rank score) is defined such that if the Mann–Whitney U-test result is significant at the 0.05 level of significance, we assign a score of 1 to district *i* at time *t*. For Late 2022, there are 16 time points, and for Early 2023, there are 17 time points. Therefore, the maximum rank score for each district 𝑖 is 16 for Late 2022 and 17 for Early 2023. Here, we defined the risk clusters as those where the rank score is greater than 1. In other words, a district with a rank score greater than 1 indicates that at least one time point was significant, indicating that the actual risk rank was higher than expected from GLM at least once. Additionally, areas where more than half of the time points within the given period are significant, resulting in a rank score is 8 or higher, are designated as the most likely clusters (MLC).

## 3 Result

### 3.1 Statistical analysis of the spatial and temporal dynamics of ASF transmission

**Figure 4:**
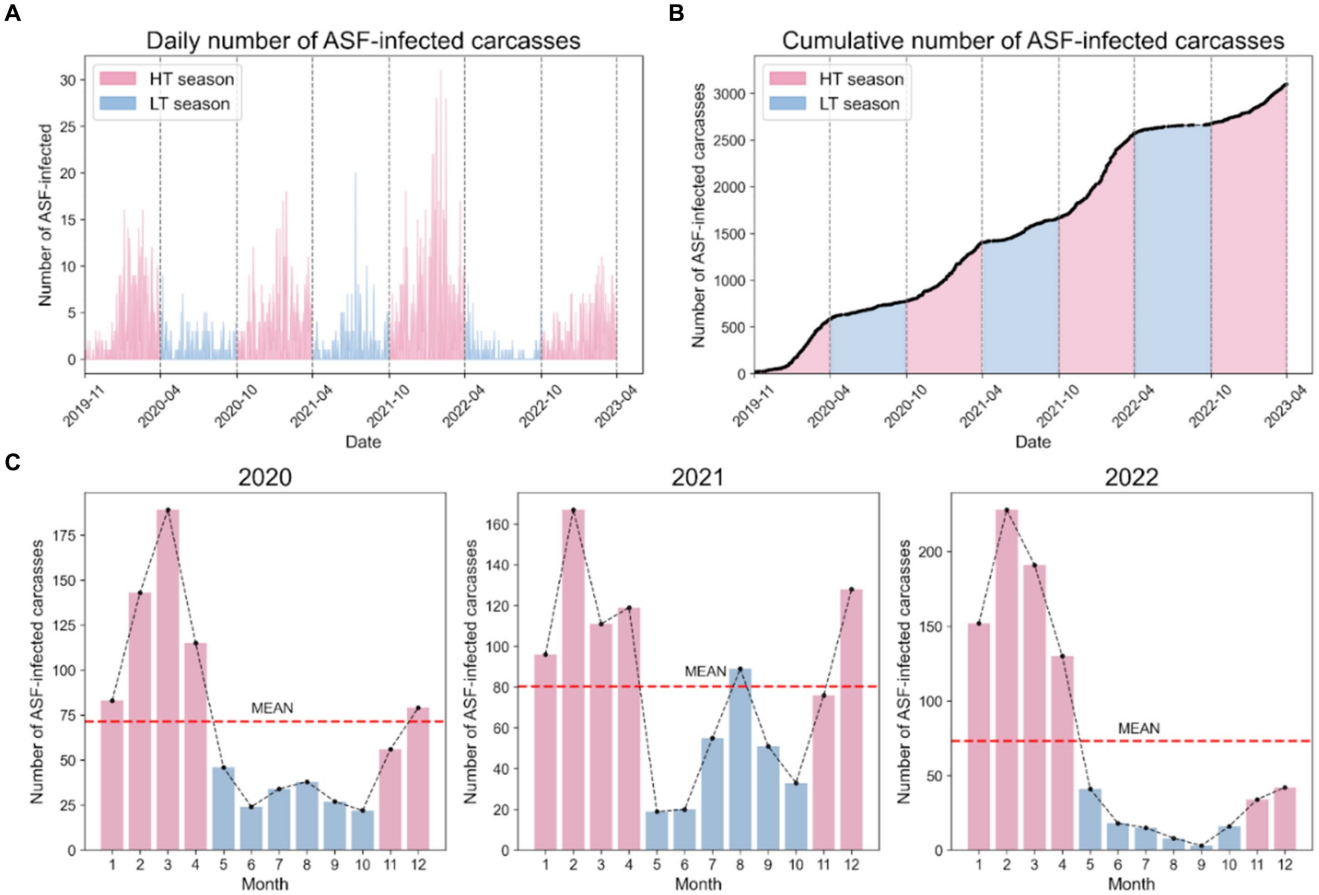
Temporal dynamics of ASF outbreaks in the Republic of Korea. **(A)** The daily cases of ASF-infected carcasses observed from 2019 to 2023. **(B)** The cumulative cases of ASF-infected carcasses observed from 2019 to 2023. **(C)** The observed instances of ASF-infected carcasses in the Republic of Korea are presented annually from 2020 to 2022. Bars in blue represent the LT season, while red bars represent the HT season. The red dashed line represents the average number of ASF-infected carcasses for that year.

Figure 4 shows the obsC data for the spatiotemporal distribution of ASF occurrences across the Republic of Korea from November 2019 to April 2023. The figure highlights the temporal dynamics of ASF outbreaks throughout the observation period, distinguishing between the HT and LT seasons to verify ASF seasonality. Figures 4A,B provide a detailed account of the daily and cumulative number of cases, reporting 2,698 ASF cases. During the HT seasons, there were 2,139 discovered carcasses, approximately 3.82 times higher than the 559 carcasses identified during the LT seasons, as shown in Figure 4A. This discrepancy is further highlighted by a more pronounced increase in cumulative cases during the HT season, as depicted in Figure 4B. Moreover, Figure 4C illustrates that the monthly instances during the LT season were significantly lower compared to the monthly averages of 71 in 2020, 80 in 2021, and 73 in 2022, emphasizing a more rapid spread of ASF throughout the Republic of Korea during the HT seasons.

The ADF test was used to evaluate the stationarity between the HT season and the LT season based on the cumulative number of ASF-infected carcasses. The ADF test yielded a *p*-value of 0.9025, exceeding the threshold of 0.05, thereby not rejecting the null hypothesis of the test, which posits that the time-series data are nonstationary. This indicates that the mean or variance of the data on the ASF spread may vary over time. Consequently, through our comprehensive analysis of seasonality, we elucidated the temporal heterogeneity in the presence of ASF in the Republic of Korea.

**Figure 5:**
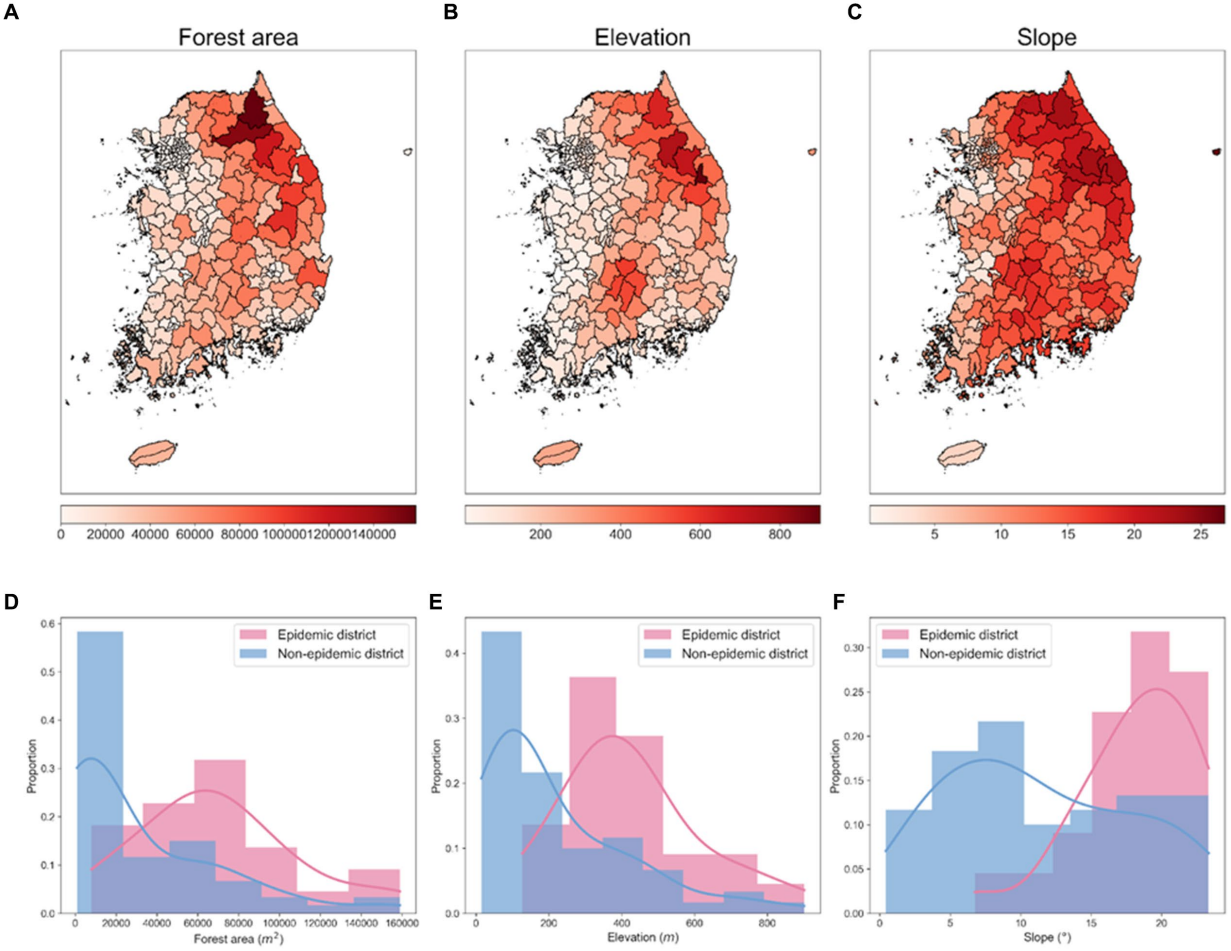
Spatial distributions of environmental factors and their proportional distributions in epidemic and non-epidemic ASF districts. **(A)** Spatial distribution of forest area (in *m^2^*). **(B)** Spatial distribution of elevation (in m). **(C)** Spatial distribution of slope (in °). Each visualization represents an area approximately 667 km vertically and 542.46 km horizontally. **(D–F)** Proportional distribution of forest area, elevation, slope in epidemic and non-epidemic districts, respectively. The shaded area represents the histogram of the data, and the solid line represents the density curve.

The environmental data used to analyze spatial characteristics and potential risk factors by administrative districts include forest area data collected from the Korea Forest Service shown in Figure 5A, and elevation and slope data extracted using QGIS (version 3.26.2) shown in Figures 5B,C. Between 2019 and 2023, 81.01% of all ASF-infected carcasses were found in the Gangwon and Gyeonggi regions in the Republic of Korea. Figures 5D–F provide a monthly comparison of the forest area, elevation, and slope data between the epidemic and non-epidemic districts within the Gyeonggi and Gangwon regions. The data shows that epidemic districts exhibited significantly higher metrics in terms of forest area, elevation, and slope compared to non-epidemic districts.

Correlation analysis further supported the geographical influence on the spread of ASF, as depicted in Supplementary Figure S1. There was a notable difference in the correlation coefficients between environmental factors and carcass counts when comparing all districts to the specific regions of Gyeonggi and Gangwon. For all districts, the correlation coefficients between carcass counts and elevation, slope, and forest area were 0.50, 0.46, and 0.48, respectively (Supplementary Figure S1A). However, these correlations were significantly higher in the Gyeonggi and Gangwon regions, with coefficients of 0.75 for elevation, 0.75 for slope, and 0.78 for forest area (Supplementary Figure S1B).

To quantify the distributional differences between epidemic and non-epidemic districts, we conducted the Ansari–Bradley test and the Mann–Whitney U test of non-parametric statistical tests. The higher *p*-values in the Ansari–Bradley test suggest variability in dispersion patterns, whereas the lower *p*-values from the Mann–Whitney U test point to concrete disparities in central tendencies. The results from the Ansari–Bradley test produced *p*-values of 0.371 for forest area, 0.095 for elevation, and 0.221 for slope, surpassing the threshold of 0.05, suggesting variance discrepancies across all environmental factors between the epidemic and non-epidemic districts. Conversely, the Mann–Whitney U test yielded *p*-values of 0.002 for forest area, 0.004 for elevation, and 0.005 for slope, all below 0.05, denoting significant distributional differences in the median values for the factors assessed. These findings highlight the distinct distributional differences of environmental factors between the epidemic and non-epidemic districts. Specifically, the forest area was highlighted as a critical factor in challenging the null hypothesis. This comprehensive analysis of environmental factors elucidated the spatial heterogeneity of ASF in the Republic of Korea and affirmed the interplay between geographical features and ASF distribution patterns.

### 3.2 Analyzing the southward trend of ASF using standard deviation ellipse

**Figure 6:**
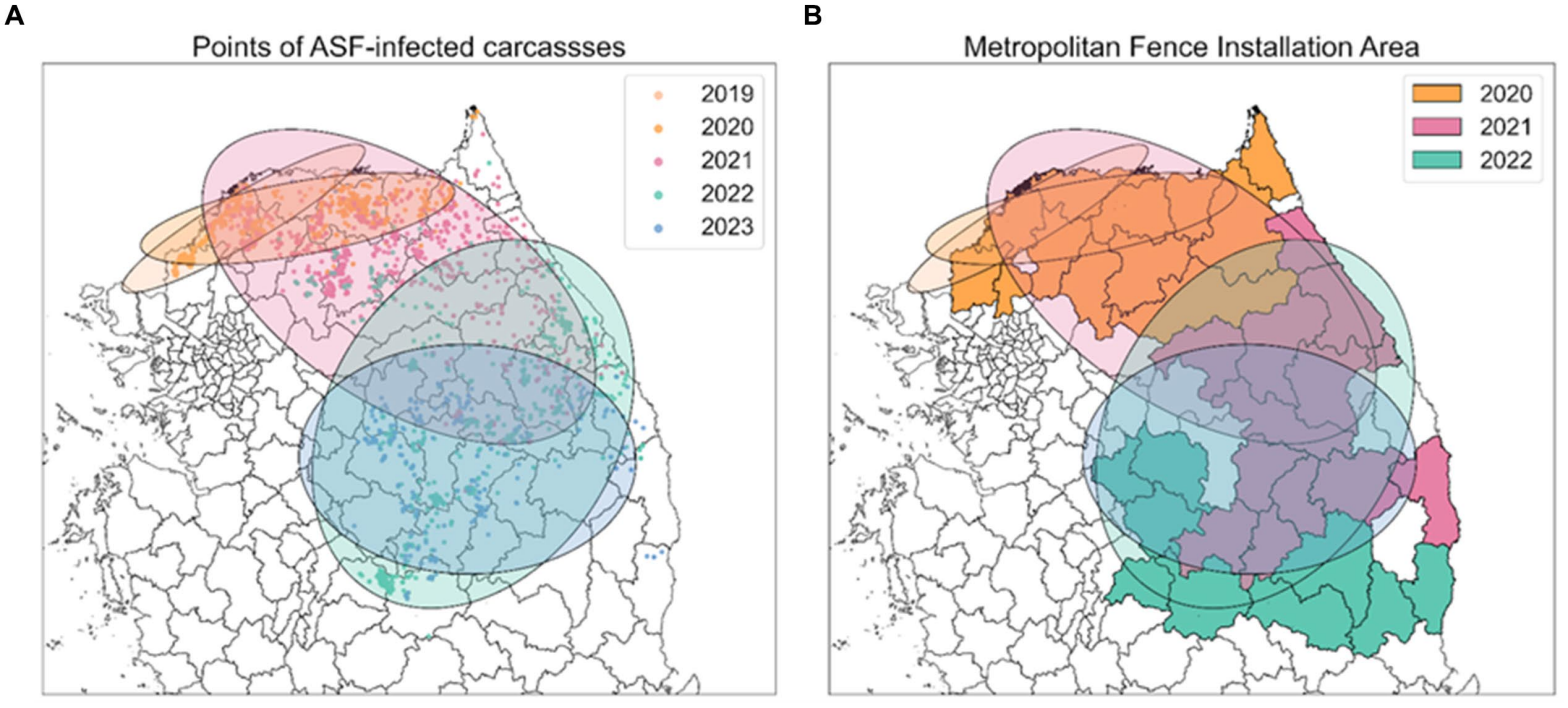
Spatial distribution of ASF-infected carcasses in the Republic of Korea. **(A)** Points indicate yearly observed ASF-infected carcasses from 2019 to 2023. **(B)** Areas where fences were newly expanded from 2020 to 2022. Red dots indicate observed ASF-infected carcasses.

Figure 6A illustrates the annual directional trend and dispersion characteristics of ASF spread from 2019 to 2023, using the SDE method. Table 1 reveals that during this period, longitude-axis changes were minimal, ranging from 2.7709 to 1.3462, while latitude-axis exhibited a significant increase, from 0.5672 to 2.4875. This indicates a notable evolution from horizontal to vertical dispersion patterns, highlighting southward shifts in the spread of ASF. Initially, in 2019, the SDE ellipse demonstrated a significant horizontal dispersion, particularly towards the northeast, as indicated by a ratio of 4.8854 and an angle of 65.4516. By 2020, this pattern had shifted more directly eastward. In subsequent years, vertical dispersion became more prominent, with ratios of 0.5412 in 2021 and 0.7654 in 2022. Specifically, in 2021, the dispersion tilted southeast (angle 109.5758), whereas in 2022, it turned towards the southwest (angle 193.6754), indicating a continuous southward movement of ASF. By 2023, the SDE ratio approached 0.9819, suggesting a nearly equal dispersion in all directions and highlighting the absence of any predominant dispersion tendency in any specific direction.

**Table 1:** Geographical information of Standard Deviation Ellipse by year.

Year	Longitude-axis	Latitude-axis	Ratio^*^	Angle	Center (latitude, longitude)
2019	2.77090	0.56721	4.8854	65.4516	(127.096, 38.111)
2020	2.51847	1.28738	1.9562	82.0699	(127.392, 38.111)
2021	1.34621	2.48751	0.5412	109.5758	(127.945, 37.809)
2022	1.71909	2.24605	0.7654	193.6745	(128.349, 37.200)
2023	1.98169	2.01814	0.9819	90.5783	(128.318, 37.043)

* Ratio indicates the longitude-to-latitude ratio.

Figure 6B displays the expansion of fencing areas from 2020 to 2022. As the discovery of ASF-infected carcasses gradually moved southward, the large-scale fencing was extended towards the south to block further spread. During the observation period, the positioning of the 2023 SDE ellipse directly above the fencing installed in 2022, indicates that these barriers effectively influenced the containment of ASF’s southward expansion. This representation highlights the strategic placement of extensive fences in response to the shifting dispersion patterns of ASF, underscoring their role in mitigating the geographical spread of ASF.

We extended the SDE analysis by incorporating its results into a GLM analysis, assuming a designated distribution to estimate the number of ASF-infected carcasses across various districts during the observation periods. As detailed in Table 2, the analysis, based on a Poisson distribution GLM, used the dataset from 2019 to 2022 as the training set, enabling a comprehensive evaluation of the variability in ASF spread across districts. The analysis revealed that the coefficient for the *Time* variable was −0.005 (i.e., $e^{-0.005}\approx 0.9950$ ), suggesting that the impact of *Time* on carcass count estimates was minimal. However, the *Season* variable showed a coefficient of approximately 1.9 ( $e^{0.648}\approx 1.9117$ ), indicating that during the HT season, the impact on carcass estimates was approximately double that of the LT season. The *Distance* variable, calculated as the negative exponent of the Euclidean distance derived from geographical coordinates, had a coefficient of 25.18. This signifies a substantial increase in carcass count as the distance decreased, confirming that closer proximity correlates with higher ASF-infected carcass detection.

**Table 2:** The result of Poisson regression.

Variables	Estimate	Standard Error	Z value	*p*-value
Intercept	−202.8000	0.2328	−87.10	<2e-16^**^
Time	−0.0050	0.0004	−11.56	<2e-16^**^
Distance	25.1800	0.7813	32.22	<2e-16^**^
Distance^2^	−16.7200	0.6452	−25.92	<2e-16^**^
Season	0.6480	0.0413	15.68	<2e-16^**^

** Signifies that the variable is statistically significant within the Poisson regression model, as indicated by the coefficient of variable yielding p-value below 0.05.

### 3.3 Identifying the risk clusters of ASF outbreaks by using rank-based method

**Figure 7:**
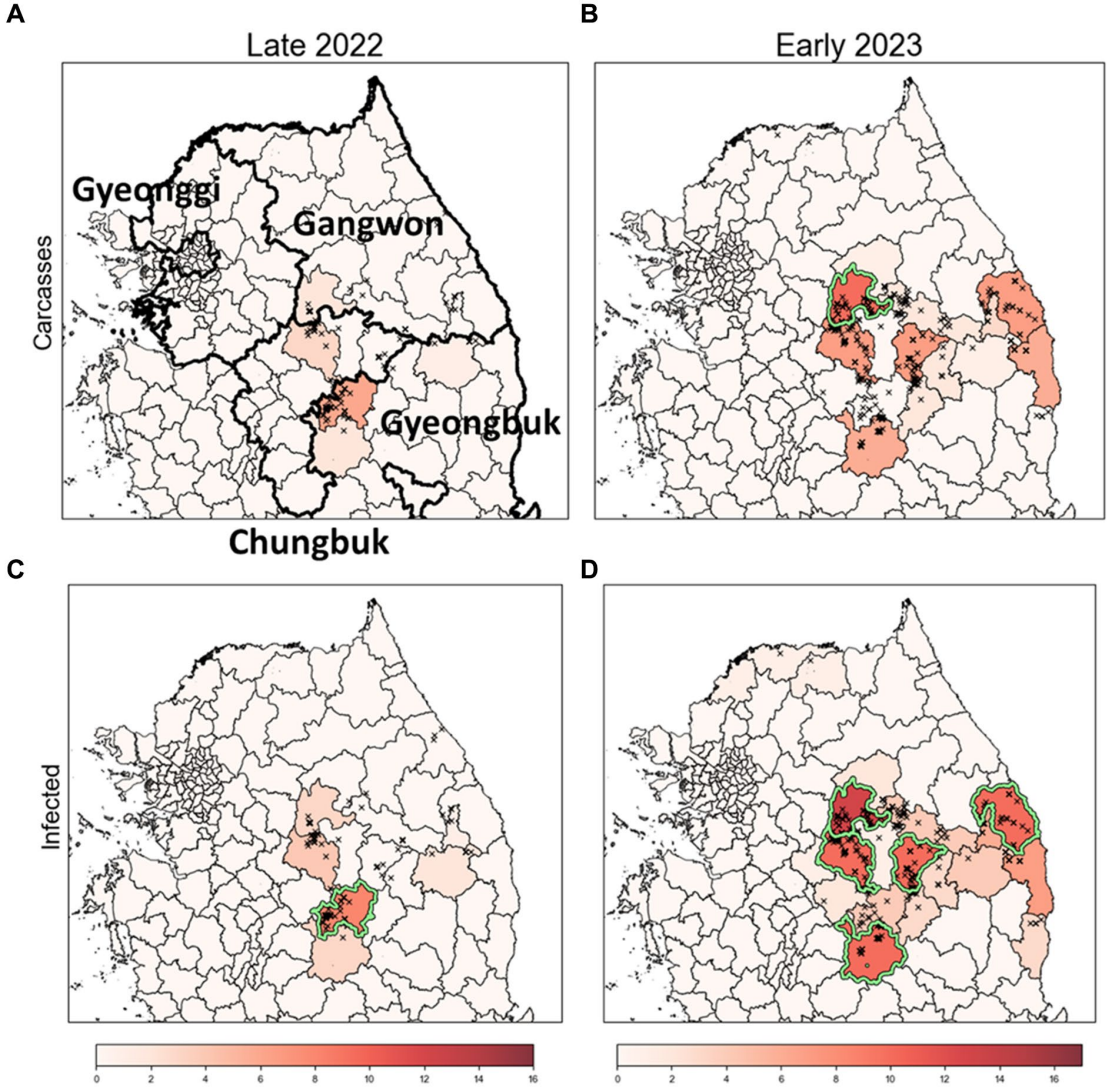
Identification of risk clusters and the most likely clusters for ASF using obsC and estI. **(A,B)** Risk clusters based on observed ASF-infected carcass data (obsC) for Late 2022 **(A)** and Early 2023 **(B)**. **(C,D)** Risk clusters based on estimated infection data (estI) for Late 2022 **(C)** and Early 2023 **(D)**. Districts marked in red indicate risk clusters, with deeper shades signifying higher rank scores. The districts with a light green colored border are identified as the most likely clusters (MLC).

Due to obsC not reflecting the delay between infection and carcass discovery, it does not accurately represent real-time infection status. To address this, we estimated the estI based on obsC. Assuming a period of 9 days from infection to death, we set the infection date prior to the observation date of obsC using a uniform distribution with a mean of 9 days. Supplementary Figure S2 illustrates the comparison between estI and obsC over time from October 2019 to April 2023. Subsequently, we applied a rank-based method incorporating the Poisson distribution to identify ASF risk clusters based on both estI and obsC, as depicted in Figure 7. This comparative approach facilitates more efficient identification of risk clusters compared to methods that rely solely on obsC.

We conducted an analysis to identify risk clusters for two distinct periods, Late 2022 and Early 2023, utilizing data from ASF-infected carcasses (Figures 7A,B) and estimates of infected individuals (Figures 7C,D). Risk clusters are marked in red on the map, with districts of higher rank scores shown in deeper shades, indicating a greater risk level. Districts achieving a rank score of 8 or above are classified as MLC and bordered with light green color. Additionally, we compared districts with high carcass counts to those based on rank scores, as detailed in Table 3 and Supplementary Figure S3.

**Table 3:** Comparison of top five risk clusters based on data for observed ASF-infected carcass counts (obsC) and estimated ASF-infected individuals (estI) with districts of high carcass counts.

Period	Districts with high carcass count	Risk clusters based on rank score using estI (Rank score)	Risk clusters based on rank score using obsC (Rank score)
Late 2022	A10. Mungyeong	A10. Mungyeong^*^ (8)	A10. Mungyeong (6)
	A6. Chungju	A6. Chungju (4)	A6. Chungju (3)
	A2. Wonju	A2. Wonju (3)	A2. Wonju (2)
	A7. Danyang	A12. Sangju (3)	A12. Sangju (2)
	A9. Bonghwa	A9. Bonghwa (2)	A19. Bonghwa (1)
Early 2023	A2. Wonju	A2. Wonju^*^ (13)	A2. Wonju^*^ (10)
	A6. Chungju	A5. Samcheok^*^ (10)	A5. Samcheok (7)
	A7. Danyang	A6. Chungju^*^ (10)	A6. Chungju (7)
	A3. Yeongwol	A7. Danyang^*^ (10)	A7. Danyang (7)
	A12. Sangju	A12. Sangju^*^ (10)	A12. Sangju (6)

A1, A2, …, A12 denote the location information for each district, as indicated in Supplementary Figure S3. (Late 2022: September to December 2022, Early 2023: January to April 2023). (∙) denotes the rank score.

* Most likely cluster.

In Late 2022, the risk clusters identified by obsC and estI were almost identical, particularly in the northwestern Gyeongbuk region, north Chungbuk region, and southwestern Gangwon region (Figures 7A,C). No districts met the MLC criteria using obsC (Figure 7A); however, based on estI, the Mungyeong district (A10) in the northwestern Gyeongbuk region was confirmed as an MLC (Figure 7C). In Early 2023, we observed a similar pattern with numerous risk clusters identified in the same regions as in Late 2022 (Figures 7B,D). Additional risk clusters were discovered in the northeastern Gyeongbuk region, northwestern Chungbuk region, and southeastern Gangwon region, leading to an expansion of identified risk clusters and MLCs. The eastward movement of wild boars in 2022 was also noted. Using obsC, only the Wonju district (A2) in the southwestern Gangwon region was identified as an MLC. In contrast, analysis using estI identified a total of five districts, including the northeastern Chungbuk region and western Gyeongbuk region, as meeting the MLC criteria. Although the identified risk clusters were generally similar between the two data sources, rank scores estimated using estI were observed to be 2–3 points higher on average than those based on obsC. This suggests that relying solely on carcass data might underestimate the actual risk level. Furthermore, although districts with high carcass counts did not always align with the risk clusters, four out of the top five districts with high carcass counts were included as top five risk clusters based on rank score in both Late 2022 and Early 2023 (Table 3). Additionally, the district with the highest incidence was classified as an MLC. These results underscore that the identified risk clusters, which require significant monitoring, consistently included districts with high incidence rates throughout the observation period.

**Figure 8:**
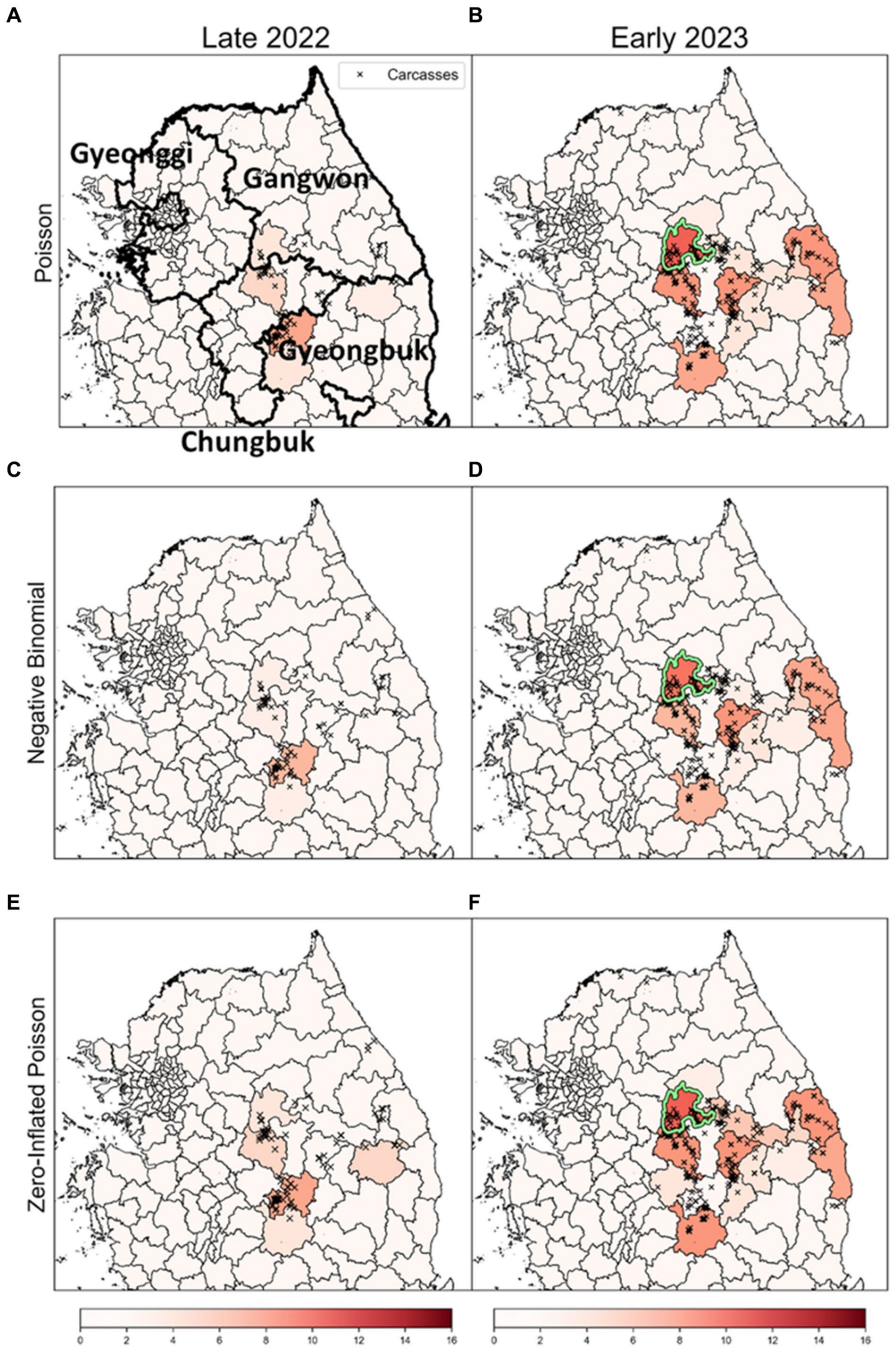
The results of a rank-based approach on different distributions. **(A,C,E)** show the results for the risk clusters from September to December 2022 (Late 2022), in row order according to each model. Similarly, **(B,D,F)** represent the estimates for the risk clusters from January to April 2023 (Early 2023), in row order according to each model. The colors of the dots signify monthly carcass counts. The districts with a light green colored border are identified as the most likely clusters (MLC).

To enhance the robustness of our analysis, we explored outcomes based on obsC across various distributions: Poisson, NB, and ZIP, as illustrated in Figure 8. Analysis for both Late 2022 and Early 2023 revealed common risk clusters across all three models, with no districts meeting the MLC criteria. However, the rank scores of districts varied among the models. Furthermore, these clusters demonstrated statistical significance based on the rank-based method, marking them as potential risk clusters for disease outbreaks and thus primary targets for surveillance. This consistency across models underscores the robustness of our risk cluster estimates. This comprehensive method of comparing results across different distribution-based models enriches our understanding of ASF outbreak dynamics. It distinctly showcases the strength of risk cluster identification derived from varied distributional assumptions and analytical methods.

**Figure 9:**
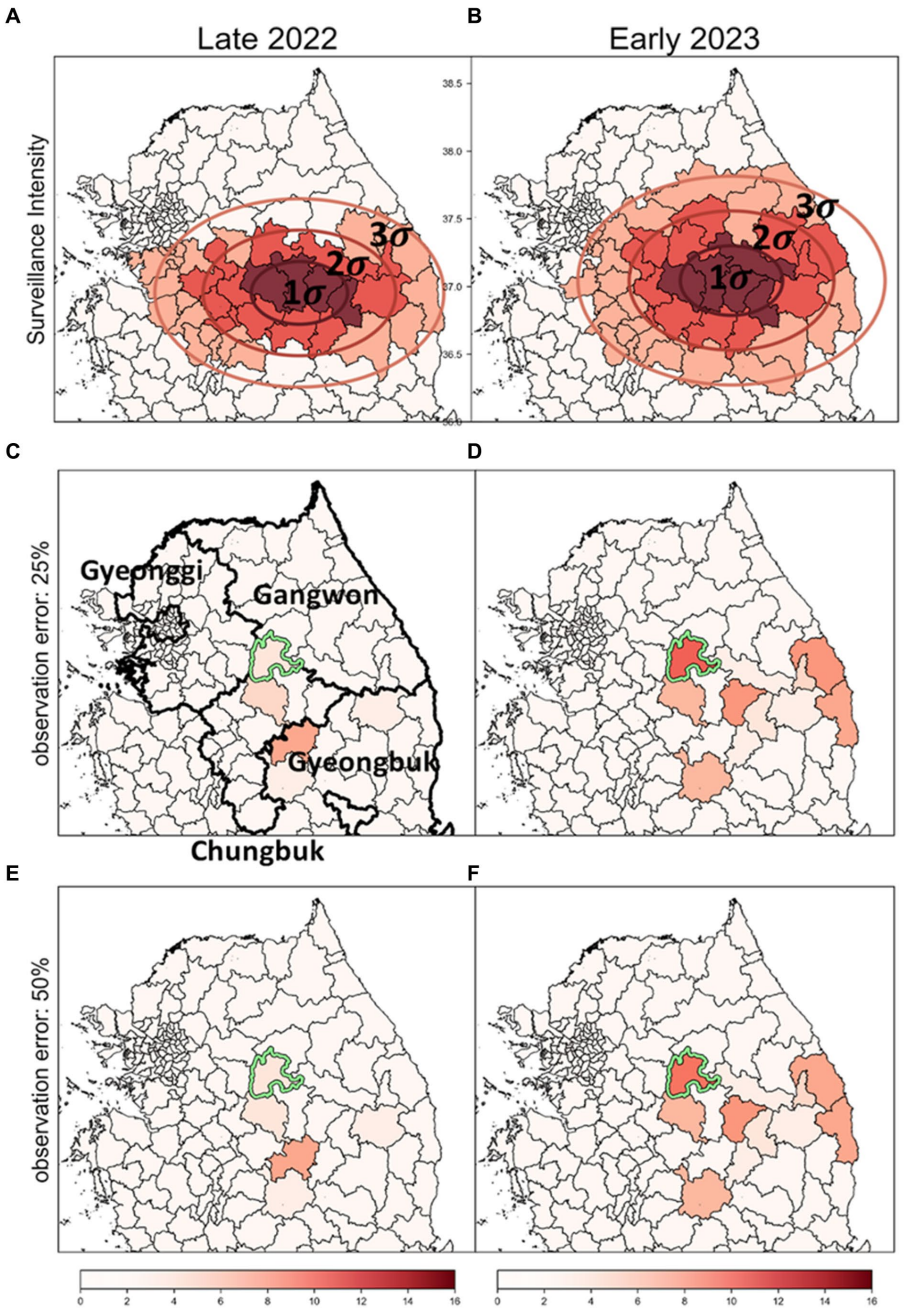
The impact of surveillance intensity and observation error on the identification of ASF risk cluster **(A,B)** Surveillance zones set up using SDE method; these zones are stratified into three levels of intensity: strong (1-sigma), intermediate (2-sigma), and low (3-sigma), to reflect the severity of ASF infection based on carcass counts. **(C,D)** Outcomes of the risk cluster when an observation error rate of 25% is applied. **(E,F)** Outcomes of the risk cluster when an observation error rate of 50% is applied. Districts marked in red indicate risk clusters, with deeper shades signifying higher rank scores. The districts with a light green colored border are identified as the most likely clusters (MLC).

In previous analyses (Figures 7, 8), it was assumed that intensities of surveillance were constant across all affected areas. However, considering the limited resources, it is plausible to adjust response intensities based on observed severity in different districts. Therefore, we designated surveillance intensity based on the concentration of discovered ASF-infected carcasses as a criterion for severity. We adjusted carcass counts accordingly and used these estimates to identify risk clusters, as depicted in Figure 9.

Surveillance zones for each period were delineated using the SDE method. The surveillance area was segmented into three zones based on sigma settings, with intensities assigned as strong, intermediate, and low (Figures 9A,B). We analyzed risk clusters and MLC with an observation error rate of 25%, as shown in Figures 9C,D, and found the results generally similar to those from analyses with uniform surveillance intensity at an observation error rate of 0% (Figures 7A,B). Even when the observation error rate was increased to 50%, hardly any significant differences were observed in the identified risk clusters (Figures 9E,F). Most districts with high carcass count and elevated risk levels were situated in areas of strong intensity (1-sigma districts), mitigating the impact of observation errors. As a result, the top-ranked risk clusters remained stable across all settings, demonstrating that the identification of risk clusters is reliable, even with considerable observation errors in districts with lower surveillance intensity.

Additionally, most of the lower-ranked risk clusters were located in districts with low intensity (3-sigma districts). The comparison of the bottom five risk clusters based on rank scores is detailed in Supplementary Table S2. Across settings with observation error rates of 0, 25, and 50%, some differences in rank scores were observed. However, during the Late 2022 period, the lower-ranked risk clusters were consistently identified across all settings. In Early 2023, four out of the five districts identified at a 0% observation error rate were also included in the bottom five risk clusters at the 25 and 50% error rates. This confirms that even significant observation errors lead to relatively minor differences in identifying lower-ranked risk clusters. Consequently, despite varying error levels, no significant differences were observed in the identification of risk clusters, underscoring the robustness of the risk identification process under different surveillance intensities and error conditions.

## 4 Discussion

The continuous nationwide spread of ASF has led to large-scale pig disposals, raising concerns within pig-farming communities and imposing significant burdens on the livestock industry, public health, and the environment. The absence of vaccinations, effective treatment methods, and the risk of infection from ASF-infected wild animals and carcasses makes controlling and preventing ASF challenging. To mitigate these challenges, the Korean government has installed broad fences as a control measure against the spread of ASF (3), though their effectiveness is limited to certain regions (34).

Understanding the spatial distribution of the spread, as informed by previous incidence data, can improve the effectiveness of containment strategies. The prevalent practice of intensive livestock farming in the Republic of Korea (25), which minimizes contact between farmed and wild animals, underscores the pivotal role of wild animal movements in the spatial transmission of ASF. We analyzed surveillance data on the carcasses of wild boars infected with ASFV in the Republic of Korea from October 2019 to April 2023. Our study aimed to analyze the spatiotemporal distribution of ASF in the Republic of Korea and identify the risk clusters of ASF transmission. We aimed to develop a statistical modeling approach to understand the spreading patterns of ASF and identify risk outbreak areas, thereby identifying crucial regions for surveillance intensities.

Initially, we used the SDE method to quantify the annual spatial dispersion and directional trends of ASF outbreaks based on their geographic locations (Figure 6). Subsequently, we developed a GLM that incorporates the distance of observed districts from the center of the ellipse, along with seasonality and time as explanatory variables. Employing a Poisson distribution, this model calculated the average number of carcasses. Subsequently, we ranked the districts based on these calculations and compared these rankings with the actual carcass counts using a rank-based method to identify risk clusters. To more accurately reflect the real-time infection status, we implemented a comparative approach, analyzing risk cluster identification based on both estI and obsC (Figure 7). Furthermore, we enhanced the robustness of our findings by comparing results derived from Poisson, ZIP, and NB distributions to ensure that our findings were not confined by the assumptions of any single distribution model (Figure 8). Given the constraints of limited resources, it is possible to adjust response intensities based on the severity observed in different districts. In response to this, we estimated adjusted carcass counts accordingly and used these estimates to identify risk clusters (Figure 9). This comprehensive approach allowed us to identify risk clusters from various perspectives, thereby enhancing the robustness of the results.

Our study revealed several key findings regarding the spread of ASF in the Republic of Korea, highlighted by three main points. First, we noted both temporal and spatial variations in the ASF outbreaks. Our analysis showed that the number of ASF cases was approximately 3.82 times higher during the spring and winter seasons (HT season) compared to the summer and autumn seasons (LT season; Figure 4). This finding aligns with previous research (53) indicating that ASF occurrences tend to be more prevalent during relatively colder seasons in the Republic of Korea. By confirming temporal heterogeneity through the ADF test, we have statistically validated that the spread of ASF varies significantly across different seasons. Furthermore, spatial analysis indicated that 81.01% of ASF occurrences were concentrated in the Gyeonggi and Gangwon regions, highlighting significant spatial disparities between epidemic and non-epidemic districts (Figure 5). This emphasizes the impact of geographical factors on the spread of ASF. Second, we observed spatial dispersion trends between 2019 and 2020, initially showing a predominantly horizontal eastward trend, then shifting to a vertical southward dispersion in 2021 and 2022. This shift, along with the trend towards a dispersion ratio close to one by 2023, suggests a potential stabilization of ASF spread southward. The dispersion patterns correlated with the habitats of wild boars and major mountain ranges in the Republic of Korea, such as the Taebaek and Sobaek Mountains (54). This suggests that the spread of ASF is influenced by natural geography and the movement of wild boars. The observed stabilization from 2022 to 2023 is speculated to result from various control policies implemented in the Republic of Korea, including the installation of extensive fencing systems (30, 54). Third, we identified risk clusters for ASF outbreaks using statistical models, including the Poisson, NB, and ZIP models. Remarkably, the identified risk clusters aligned with the geographic locations of the Republic of Korea’s major mountain ranges, as corroborated by spatial dispersion trends. Additionally, we estimated the number of infected individuals to aid in identifying risk clusters. While there were no significant differences in the districts designated as risk clusters, the number of districts classified as MLC differed. Furthermore, we compared the identification of risk clusters based on adjusted ASF-infected carcass counts across varying observation error rates (0, 25, 50%). The results confirmed that risk cluster identification exhibited no significant differences across all scenarios. The findings provide insights to enhance the effectiveness of surveillance strategies and control measures. Specifically, they suggest the potential for developing customized preventive strategies based on seasonal and regional variations, thereby improving the efficiency of resource allocation for disease control and providing evidence for targeted intensive control measures to effectively suppress the spread of ASF by identifying risk clusters. These insights underscore the utility of statistical models in enhancing preemptive actions such as wildlife population control, carcass removal, and the installation of extensive fencing, all of which are currently implemented in the country.

This study has several limitations. Due to the challenges in collecting real-time data on ASF-infected wild boars, our analysis was restricted to surveillance data derived from wild boar carcasses, as observed in previous studies (36, 37). This reliance on surveillance data may lead to potential risk underestimation compared to actual occurrences. Therefore, our approach focused on understanding dispersion trends and identifying potential risk clusters rather than predicting the exact number of carcasses in real time. Unlike previous studies that have largely focused on predicting risk clusters rather than estimating carcass numbers (53), our research incorporates environmental factors, such as forest area, distinguishes time intervals into HT and LT seasons, and analyzes risk clusters, marking a novel approach in the field.

Furthermore, the inability to determine the exact moment of infection based on the timing and location data of the carcasses limits precise outbreak analysis. Recognizing these constraints, previous research has concentrated on assessing the risk of infection. However, by applying the GLM and rank-based score methods, our study goes beyond merely identifying regions with a high number of carcasses by pinpointing areas with significantly higher occurrences than average, based on the discrepancy between actual data and model estimates. The application of various probability distributions, including Poisson, NB, and ZIP distributions, to identify risk clusters demonstrates the importance of a comprehensive analysis for understanding ASF dynamics. Additionally, we estimated ASF-infected cases from surveillance data and compared these with identified high risk and caution areas to verify underestimation due to risk estimation based on ASF-infected carcasses. This approach highlights the significance of identifying areas with significantly higher risk potential than expected, offering a nuanced understanding of ASF spread, and contributing to more effective disease management and prevention strategies.

Another point is the enhancement of efficiency and accuracy in identifying risk clusters through the application of artificial intelligence (AI) methods. In the previous study by Orusa et al. (55), the use of AI methods in conjunction with GIS and remote sensing technologies demonstrated the potential of geospatial deep learning AI to process complex datasets and provide actionable insights for wildlife disease monitoring and management. By leveraging AI, it is possible to develop more robust and adaptable disease management strategies that can respond to the evolving dynamics of diseases such as ASF. This integration enhances the efficiency and accuracy of identifying risk clusters, thereby improving the effectiveness of targeted surveillance and control measures. Such an approach will be instrumental in advancing our understanding of ASF dynamics and enhancing our ability to implement timely and effective interventions.

Despite these limitations, the application of multiple statistical models and the integration of environmental factors, such as forest areas and seasonal variations, mark an alternative approach in the field. Our findings underscore the significant influence of temporal and spatial heterogeneity on the spread of ASF in the Republic of Korea, highlighting the intricate relationship between ASF dynamics, geographical features, and the role of wild boar habitats and movement patterns. Identifying risk clusters for targeted surveillance and control measures is crucial, contributing to more effective disease management and prevention strategies. This study offers insights into the strategic planning of surveillance and control measures, aiming for a more targeted approach for managing ASF outbreaks.

## 5 Conclusion

This study analyzed the spatial and temporal heterogeneity of ASF in the Republic of Korea using surveillance data and revealed an annual southward propagation pattern. The angles of the SDE ellipse in 2021 and 2022, denoted as 109.5758 and 193.6745, respectively, indicate an annual southward propagation pattern. However, the ratio of the SDE ellipse in 2023, which was 0.9819, indicates that the southward movement was suppressed in 2023. This could be interpreted as an effect of measures such as the installation of extensive fences in certain areas during that year. We introduced a new statistical model that allowed us to predict the average monthly number of carcasses per district. We successfully identified risk clusters with significantly higher ranks based on observed ASF-infected carcasses compared to areas with high ranks based on the estimated ASF-infected carcasses. This study contributes significantly to the epidemiology and dynamics of animal infectious diseases by emphasizing the importance of understanding spatially concentrated risk clusters. By providing crucial data for the efficient allocation of disease management and preventive measures, this study lays the foundation for improving ASF management strategies.

## Data availability statement

The original contributions presented in the study are included in the article/Supplementary material, further inquiries can be directed to the corresponding authors.

## Author contributions

KK: Visualization, Validation, Formal analysis, Data curation, Writing – original draft, Methodology. JO: Writing – original draft, Visualization, Formal analysis, Data curation. YC: Writing – original draft, Validation, Methodology, Conceptualization. HL: Writing – review & editing, Writing – original draft, Supervision, Methodology, Funding acquisition, Conceptualization. CS: Writing – review & editing, Methodology, Visualization.

## References

[1] Galindo I Alonso C. African swine fever virus: a review. Viruses. (2017) 9:103. doi: 10.3390/v9050103, PMID: 28489063 PMC5454416

[2] World Organization for Animal Health (2024). Available at: https://www.woah.org/en/disease/african-swine-fever/ [Accessed March 8, 2024].

[3] Jo YS Gortázar C. African swine fever in wild boar, South Korea, 2019. Transbound Emerg Dis. (2020) 68:2878–89. doi: 10.1111/tbed.13532 33844467

[4] Quarantine Information Agency, Republic of Korea (2024). Causes and Transmission Methods of African Swine Fever. Available at: https://www.qia.go.kr/animal/prevent/ani_africa_pig_fever_germ.jsp [Accessed March 8, 2024].

[5] Viani A Orusa T Borgogno-Mondino E Orusa R. Snow metrics as proxy to assess sarcoptic mange in wild boar: preliminary results in Aosta Valley (Italy). Life. (2023) 13:987. doi: 10.3390/life13040987, PMID: 37109516 PMC10143256

[6] Orusa T Viani A Moyo B Cammareri D Borgogno-Mondino E. Risk assessment of rising temperatures using Landsat 4–9 LST time series and Meta® population dataset: An application in Aosta Valley, NW Italy. Remote Sens. (2023) 15:2348. doi: 10.3390/rs15092348

[7] Carella E Orusa T Viani A Meloni D Borgogno-Mondino E Orusa R. An integrated, tentative remote-sensing approach based on NDVI entropy to model canine distemper virus in wildlife and to prompt science-based management policies. Animals. (2022) 12:1049. doi: 10.3390/ani12081049, PMID: 35454295 PMC9029328

[8] Orusa T Orusa R Viani A Carella E Borgogno ME. Geomatics and EO data to support wildlife diseases assessment at landscape level: a pilot experience to map infectious keratoconjunctivitis in chamois and phenological trends in Aosta Valley (NW Italy). Remote Sens. (2020) 12:3542. doi: 10.3390/rs12213542

[9] Montgomery RE. On a form of swine fever occurring in British East Africa (Kenya Colony). J Comp Pathol. (1921) 34:159–91. doi: 10.1016/S0368-1742(21)80031-4

[10] EFSA Panel on Animal Health and Welfare (AHAW). Scientific opinion on African swine fever. EFSA J. (2010) 8:1556. doi: 10.2903/j.efsa.2010.1556

[11] Zhou X Li N Luo Y Liu YE Miao F Chen T. Emergence of African swine fever in China, 2018. Transbound Emerg Dis. (2018) 65:1482–4. doi: 10.1111/tbed.12989, PMID: 30102848

[12] Denstedt E Porco A Hwang J Nga NT Ngoc PT Chea S. Detection of African swine fever virus in free-ranging wild boar in Southeast Asia. Transbound Emerg Dis. (2021) 68:2669–75. doi: 10.1111/tbed.13964, PMID: 33351995 PMC8518571

[13] World Organization for Animal Health (2024). African Swine Fever in Asia. Available at: https://rr-asia.woah.org/en/projects/asf/ [Accessed May 30, 2024].

[14] Food and Agriculture Organization of the United Nations (2024). African swine fever (ASF) situation update in Asia & Pacific. Available at: https://www.fao.org/animal-health/situation-updates/asf-in-asia-pacific/en [Accessed March 8, 2024].

[15] Korea Animal Health Information System (2024). Swine fever causative agent. Available at: https://home.kahis.go.kr/home/lkdissinfo/ani_m2_02.do [Accessed March 8, 2024].

[16] O’Neill X White A Ruiz-Fons F Gortázar C. Modeling the transmission and persistence of African swine fever in wild boar in contrasting European scenarios. Sci Rep. (2020) 10:5895. doi: 10.1038/s41598-020-62736-y, PMID: 32246098 PMC7125206

[17] Ministry of Environment, Republic of Korea (2024). Improvement measures for African Swine Fever (ASF) in wild boars. Available at: https://www.me.go.kr/home/web/board/read.do?menuId=10525&boardMasterId=1&boardCategoryId=39&boardId=1672720 [Accessed May 30, 2024].

[18] Kim HJ Cho KH Lee SK Kim DY Nah JJ Kim HJ. Outbreak of African swine fever in South Korea, 2019. Transbound Emerg Dis. (2020) 67:473–5. doi: 10.1111/tbed.13483 31955520

[19] Ministry of Environment, Republic of Korea (2024). Current status of wild boar ASF (African swine fever) outbreak in Korea. ASF Updates. Available at: https://www.me.go.kr/home/web/index.do?menuId=10259 [Accessed March 8, 2024].

[20] Ministry of Agriculture, Food and Rural Affairs, Republic of Korea (2024). Information on the outbreak of livestock infectious disease (ASF). Available at https://www.mafra.go.kr/FMD-AI2/2145/subview.do [Accessed March 8, 2024].

[21] Ito S Bosch J Martínez-Avilés M Sánchez-Vizcaíno JM. The evolution of African swine fever in China: a global threat? Front Vet Sci. (2022) 9:828498. doi: 10.3389/fvets.2022.828498, PMID: 35425825 PMC9001964

[22] Kim G Park JE Kim SJ Kim Y Kim W Kim YK. Complete genome analysis of the African swine fever virus isolated from a wild boar responsible for the first viral outbreak in Korea, 2019. Front Vet Sci. (2023) 9:1080397. doi: 10.3389/fvets.2022.1080397, PMID: 36713858 PMC9875005

[23] Cho KH Hong SK Kim DY Jang MK Kim JH Lee H. Pathogenicity and pathological characteristics of African swine fever virus strains from pig farms in South Korea from 2022 to January 2023. Pathogens. (2023) 12:1158. doi: 10.3390/pathogens12091158, PMID: 37764966 PMC10534632

[24] Lim JS Vergne T Pak SI Kim E. Modelling the spatial distribution of ASF-positive wild boar carcasses in South Korea using 2019–2020 national surveillance data. Animals. (2021) 11:1208. doi: 10.3390/ani11051208, PMID: 33922261 PMC8145688

[25] Na IJ. Trends in domestic animal welfare policy. Korea Rural Economic Institute. (2014) 163:91–103. doi: 10.1002/9781118329689

[26] Boklund A Dhollander S Chesnoiu Vasile T Abrahantes JC Bøtner A Gogin A. Risk factors for African swine fever incursion in Romanian domestic farms during 2019. Sci Rep. (2020) 10:10215. doi: 10.1038/s41598-020-66381-3, PMID: 32576841 PMC7311386

[27] Hone J. Applied population and community ecology: The case of feral pigs in Australia. UK: John Wiley & Sons (2012). 200 p.

[28] European Food Safety Authority. Evaluation of possible mitigation measures to prevent introduction and spread of African swine fever virus through wild boar. EFSA J. (2014) 12:3616. doi: 10.2903/j.efsa.2014.3616

[29] European Food Safety Authority (EFSA) Boklund A Cay B Depner K Földi Z Guberti V. Epidemiological analyses of African swine fever in the European Union (November 2017 until November 2018). EFSA J. (2018) 16:e05494. doi: 10.2903/j.efsa.2018.5494, 32625771 PMC7009685

[30] Maximizing Efforts to Prevent African Swine Fever in Wild Boars in the Gyeongbuk Region (2023). Available at: https://www.korea.kr/briefing/pressReleaseView.do?newsId=156588906 [Accessed March 27, 2024].

[31] Yonhap News (2024). Available at: https://www.yna.co.kr/view/AKR20240321139800530 [Accessed May 30, 2024].

[32] Lim JS Andraud M Kim E Vergne T. Three years of African swine fever in South Korea (2019–2021): a scoping review of epidemiological understanding. Transbound Emerg Dis. (2023) 2023:1–15. doi: 10.1155/2023/4686980 PMC12016690 40303829

[33] Dellicour S Desmecht D Paternostre J Malengreaux C Licoppe A Gilbert M. Unravelling the dispersal dynamics and ecological drivers of the African swine fever outbreak in Belgium. J Appl Ecol. (2020) 57:1619–29. doi: 10.1111/1365-2664.13649

[34] Han J Yoo D Pak S. Understanding the transmission of African swine fever in wild boars of South Korea: a simulation study for parameter estimation. Transbound Emerg Dis. (2022) 69:e1101–12. doi: 10.1111/tbed.14403 34821474

[35] ASF Real-Time Status Plate. PigPeople (2023) Available at: 0http://www.pigpeople.net/news/article.html?no=12681 [Accessed March 8, 2024]

[36] Loi F Di Sabatino D Baldi I Rolesu S Gervasi V Guberti V. Estimation of R0 for the spread of the first ASF epidemic in Italy from fresh carcasses. Viruses. (2022) 14:2240. doi: 10.3390/v14102240, PMID: 36298795 PMC9607429

[37] Gervasi V Guberti V. African swine fever endemic persistence in wild boar populations: key mechanisms explored through modelling. Transbound Emerg Dis. (2021) 68:2812–25. doi: 10.1111/tbed.14194, PMID: 34255414 PMC9292501

[38] Yoon H Son Y Kim K-S Lee I Kim Y-H Em L. Estimating the time of infection for African swine fever in pig farms in Korea. Front Vet Sci. (2023) 10:10. doi: 10.3389/fvets.2023.1281152, PMID: 38076564 PMC10701385

[39] Lee SM. Reproductive performance and sex ratio adjustment of the wild boar (*Sus scrofa*) in South Korea. Sci Rep. (2022) 12:21774. doi: 10.1038/s41598-022-25626-z, PMID: 36526656 PMC9758127

[40] Mushtaq R. (2011). Augmented dickey fuller test.

[41] Ansari AR Bradley RA. Rank-sum tests for dispersions. Ann Math Stat. (1960) 31:1174–89. doi: 10.1214/aoms/1177705688

[42] McKnight PE Najab J. (2010). Mann-Whitney U test. The Corsini encyclopedia of psychology.

[43] Yuill RS. The standard deviational ellipse; an updated tool for spatial description. Geogr Ann Ser B, Hum Geogr. (1971) 53:28–39. doi: 10.1080/04353684.1971.11879353

[44] Gong J. Clarifying the standard deviational ellipse. Geogr Anal. (2002) 34:155–67. doi: 10.1111/j.1538-4632.2002.tb01082.x

[45] Lu Y Deng X Chen J Wang J Chen Q Niu B. Risk analysis of African swine fever in Poland based on spatio-temporal pattern and Latin hypercube sampling, 2014–2017. BMC Vet Res. (2019) 15:160. doi: 10.1186/s12917-019-1903-z, PMID: 31118049 PMC6532167

[46] Chen J Wang J Wang M Liang R Lu Y Zhang Q. Retrospect and risk analysis of foot-and-mouth disease in China based on integrated surveillance and spatial analysis tools. Front Vet Sci. (2020) 6:511. doi: 10.3389/fvets.2019.00511, PMID: 32039251 PMC6986238

[47] Ebdon D. Statistics in geography. 2nd ed. Malden, MA: Blackwell Publishers Ltd (1985).

[48] Moore TW McGuire MP. Using the standard deviational ellipse to document changes to the spatial dispersion of seasonal tornado activity in the United States. NPJ Clim Atmos Sc. (2019) 2:21. doi: 10.1038/s41612-019-0078-4

[49] Wang B Wenzhong S Zelang M. Confidence analysis of standard deviational ellipse and its extension into higher dimensional Euclidean space. PloS one. (2015) 10:e0118537. doi: 10.1371/journal.pone.0118537, PMID: 25769048 PMC4358977

[50] Kulldorff M. A spatial scan statistic. Commun Stat Theor M. (2007) 26:1481–96. doi: 10.1080/03610929708831995

[51] Loi F Cappai S Coccollone A Rolesu S. Standardized risk analysis approach aimed to evaluate the last African swine fever eradication program performance, in Sardinia. Front Vet Sci. (2019) 6:299. doi: 10.3389/fvets.2019.00299, PMID: 31572734 PMC6753231

[52] Rosychuk RJ Chang H. A spatial scan statistic for compound Poisson data. Stat Med. (2013) 32:5106–18. doi: 10.1002/sim.5891, PMID: 23824973

[53] Lim SJ Namgung H Kim NH Oh Y Park YC. Prediction of potential spread areas of African swine fever virus through wild boars using the Maxent model. J Ecol Environ. (2022) 46:46. doi: 10.5141/jee.22.006

[54] Choi SK Chun S An J Lee MY Kim HJ Min MS. Genetic diversity and population structure of the long-tailed goral, *Naemorhedus caudatus*, in South Korea. Genes Genet Sys. (2015) 90:31–41. doi: 10.1266/ggs.90.31, PMID: 26119664

[55] Orusa T Viani A Borgogno-Mondino E. Earth observation data and geospatial deep learning ai to assign contributions to European municipalities Sen 4MUN: an empirical application in Aosta Valley (NW Italy). Land. (2024) 13:80. doi: 10.3390/land13010080

