# Supplementary material for: Identifying risk clusters for African swine fever in Korea by developing statistical models
Source: Front Vet Sci. 2024 Jul 24;11:1416862. doi: 10.3389/fvets.2024.1416862 (PMC11303289; doi:10.3389/fvets.2024.1416862)
Supplement: Supplementary file 1 [file Data_Sheet_1.docx]

**Table S1. Summary of the surveillance data and environmental data.** The forest area percentage (%) was calculated by averaging the forest-to-land ratios of municipalities within each region. The slope and elevation were determined by averaging the municipal values within each region. The ASF-positive wild boar carcass count was based on yearly and seasonal (HT: Nov-Apr, LT: May-Oct) averages.

|  | | **Gyeonggi** | **Gangwon** | **Republic of Korea** |
| --- | --- | --- | --- | --- |
| **Forest area (%)** | | 50.23 | 81.2 | 62.72 |
| **Slope (°)** | | 7.87 | 19.38 | 10.7 |
| **Altitude (m)** | | 108.12 | 472.68 | 165.85 |
| **Carcasses count** | | 675 | 1834 | 3097 |
| **2019** | HT | 26 | 11 | 37 |
|  | LT | 12 | 6 | 18 |
| **2020** | HT | 334 | 331 | 665 |
|  | LT | 77 | 114 | 191 |
| **2021** | HT | 177 | 467 | 697 |
|  | LT | 28 | 239 | 267 |
| **2022** | HT | 15 | 482 | 777 |
|  | LT | 6 | 48 | 101 |
| **2023** | HT | 0 | 136 | 344 |


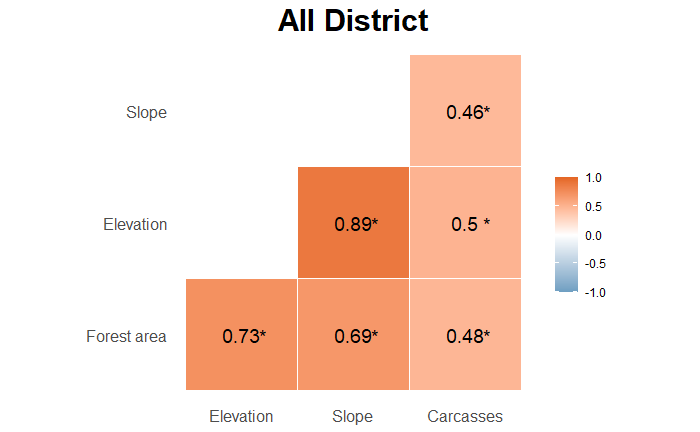

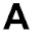

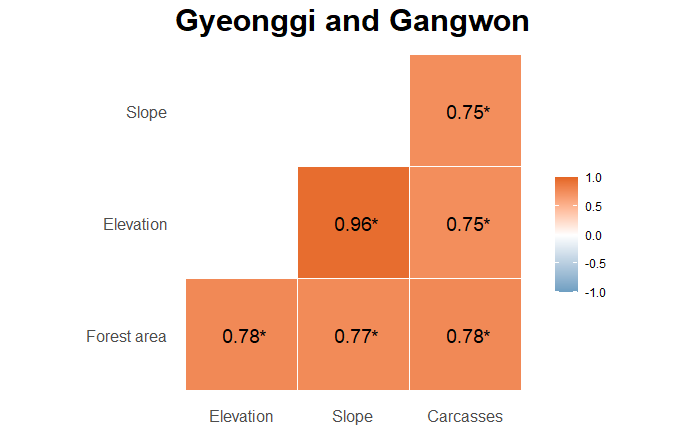

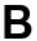


**Figure S1. Correlation matrices illustrating the relationships between carcass counts and environmental factors in all districts vs. Gyeonggi and Gangwon Regions** (A) All districts, and (B) Gyeonggi and Gangwon regions. The correlation coefficients indicate a stronger relationship between carcass counts and these environmental factors in the Gyeonggi and Gangwon regions, with particularly high correlations observed for elevation (0.75), slope (0.75), and forest area (0.78).


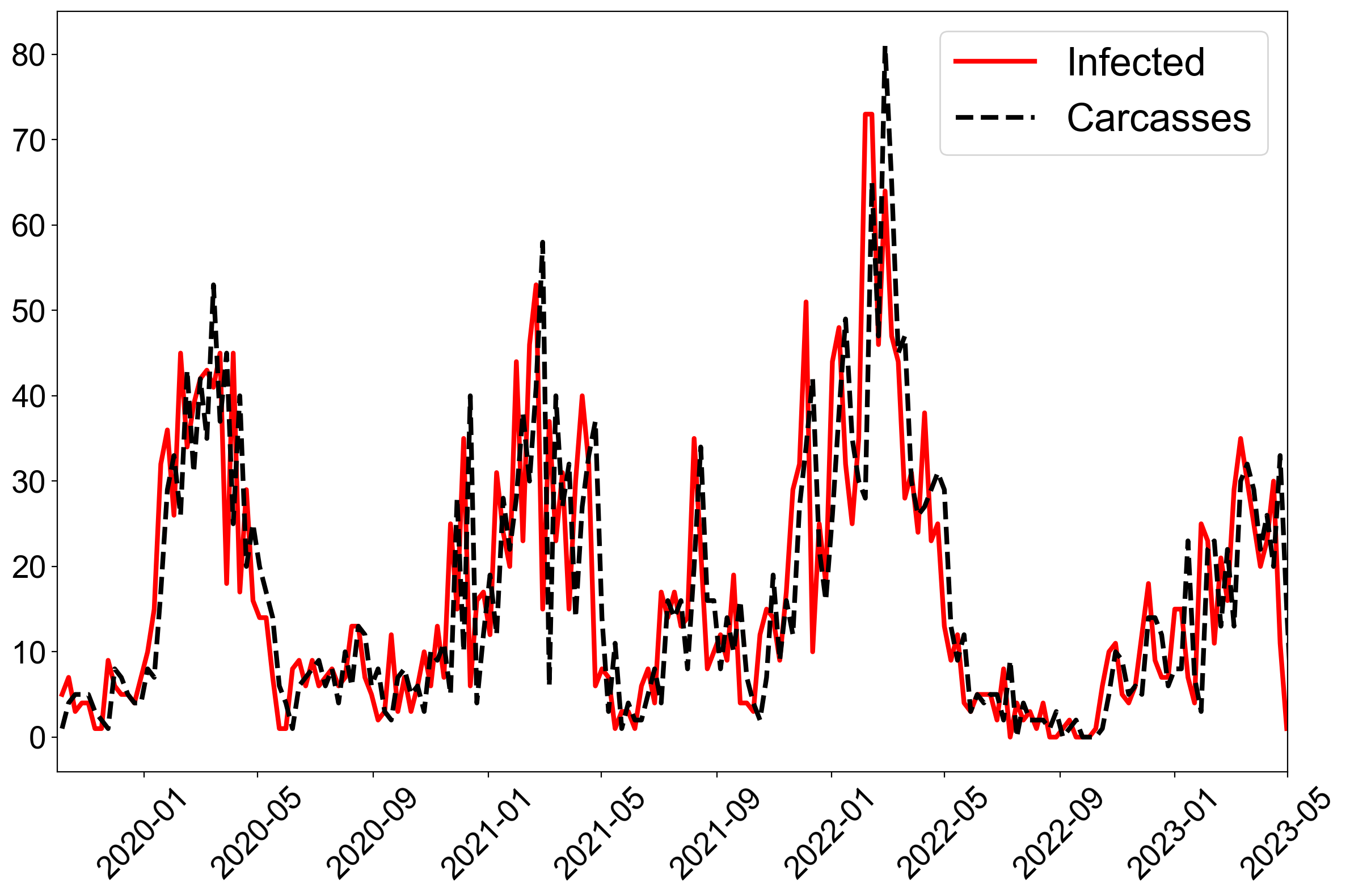


**Figure S2. Estimation of ASF-infected individuals (estI) through back-calculation method with uniform distribution.** The black dashed line represents obsC and red solid line indicates the estimated estI using obsC from October 2019 to May 2023.


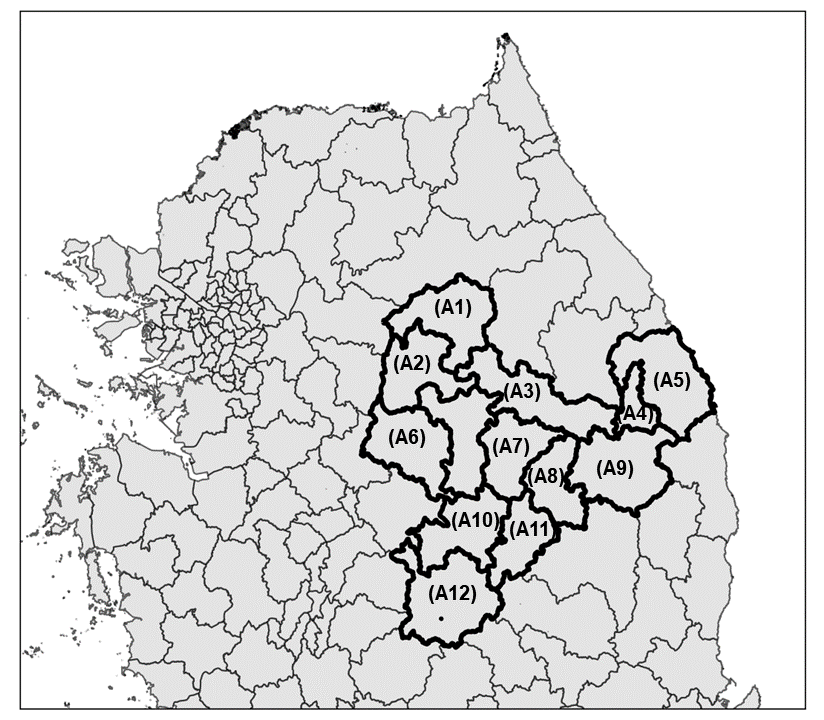


**Figure S3. Districts with the top five rank scores or bottom five rank scores.** (A1): Hoengsung, (A2): Wonju, (A3): Yeongwol, (A4): Taebaek, (A5): Samcheok, (A6): Chungju, (A7): Danyang, (A8): Yeongju, (A9): Bonghwa, (A10): Mungyeong, (A11): Yecheon, (A12): Sangju.

**Table S2. The bottom five low risk districts according to surveillance intensity.**

| **Observation error rate** | **0%** | **25%** | **50%** |
| --- | --- | --- | --- |
| **Late 2022** | A10. Mungyeong (6) | A10. Mungyeong (6) | A10. Mungyeong (6) |
|  | A6. Chungju (3) | A6. Chungju (3) | A6. Chungju (2) |
|  | A2. Wonju (2) | A2. Wonju (2) | A2. Wonju (2) |
|  | A12. Sangju (2) | A12. Sangju (1) | A12. Sangju (1) |
|  | A9. Bonghwa (1) | A9. Bonghwa (1) | A9. Bonghwa (1) |
| **Early 2023** | A3. Yeongwol (2) | A4. Taebaek (3) | A4. Taebaek (3) |
|  | A8. Yeongju (2) | A8. Yeongju (2) | A8. Yeongju (2) |
|  | A11. Yecheon (2) | A3. Yeongwol (1) | A3. Yeongwol (1) |
|  | A9. Bonghwa (1) | A11. Yecheon (1) | A11. Yecheon (1) |
|  | A1. Hoengseong (1) | A9. Bonghwa (1) | A9. Bonghwa (1) |

A1, A2, … , A12 denote the location information for each district, as indicated in Figure S3. (Late 2022: September to December 2022, Early 2023: January to April 2023).

(∙) denotes the rank score.
